# The Anion–Cation Relay Battery Prototype

**DOI:** 10.1002/smsc.202000030

**Published:** 2020-11-12

**Authors:** Huawei Song, Jian Su, Chengxin Wang

**Affiliations:** ^1^ State Key Laboratory of Optoelectronic Materials and Technologies School of Materials Science and Engineering Sun Yat‐sen (Zhongshan) University Guangzhou 510275 P. R. China

**Keywords:** anion–cation, high energy/power, membranes, rechargeable batteries, relay insertion/extraction

## Abstract

Relay insertion/extraction chemistry of both anions and cations on the cathode is disclosed for non‐aqueous rechargeable batteries, different from previous metal‐ion batteries (MIBs) and dual‐ion batteries (DIBs) of only positively or negatively charged ions. The “anion–cation relay battery (ACRB)” fully uses both negative and positive ions and offers bright prospects for high‐specific‐energy and large‐rate grid scale energy storage. Proof‐of‐concept ACRBs with commercial Li/Na/K plate as anodes and free‐standing few‐layered graphitic carbon (FLGC) membrane as cathodes demonstrate impressive overall cell performance (a reversible capacity of ≈300 mAh g^−1^ at 100 mA g^−1^, service life >23 000 cycles with a retention decay of ≈0.0013% per cycle, and a cathode energy density of ≈370 Wh kg^−1^ at ≈27 kW kg^−1^), comparable to the highest level counterparts. The work may set a promising strategy to break the predicament facing by various MIBs and DIBs, and also a direction to forward cost cutting in commercial lithium‐ion batteries (LIBs).

## Graphical abstract

The basic concepts and working mechanism of anion–cation relay battery prototype are established. This type of battery offers bright prospects for high‐specific‐energy and large‐rate grid scale energy storage by relay insertion/extraction chemistry of both anions and cations on the cathode. The exemplified batteries demonstrate impressive overall performance, comparable to the most advanced counterparts.

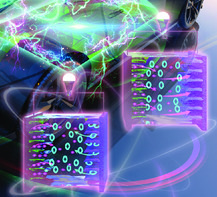

## 1 Introduction

Cation insertion reaction has contributed huge progress in various rechargeable metal‐ion battery (MIB) technologies in recent years.^[^ ^1^, ^2^, ^3^ ^]^ Among them, the graphite/LiCoO_2_ cell, prototype of current lithium‐ion battery (LIB), works through Li‐ion intercalation/de‐intercalation at the cathode and anode, delivering an energy density of ≈120–160 Wh kg^−1^.^[^ ^4^, ^5^, ^6^ ^]^ Due to the limited Li‐ion storage capability and high cost of cobalt resource, afterward, LiFePO_4_, ternary LiNi_*x*_Co_*y*_Mn_1−*x*−*y*_O_2_ (NCM), and Li‐rich manganates as more promising candidates for the cathodes, along with Si‐ or Sn‐based anodes with larger capacity due to conversion and alloying reaction of multielectron transfer, have been introduced, and the new combinations have pushed the benchmark up to ≈220–250 Wh kg^−1^.^[^ ^4^, ^7^, ^8^ ^]^ Simultaneously, nanoscale material design, rational fabrication of multidimensional ion‐/electron pathway, and surface/interface and defect engineering enable fast kinetics and superior power performance due to largely shortened Li‐diffusion depth and smooth transfer of charge carriers, even resulting into considerable capacitive energy storage working on the timescale of a few seconds.^[^ ^9^, ^10^, ^11^ ^]^ The representative research work of Tolbert and co‐workers and Dunn and co‐workers concluded that the Li‐storage power performance of MoO_3_ could be significantly enhanced by ordered mesopores in iso‐oriented nanocrystalline walls and oxygen vacancies due to increased redox pseudocapacitance, and Duan and co‐workers substantiated that highly interconnected graphene network in 3D holy‐graphene/Nb_2_O_5_ nanoarchitecture promoted the electron/ion transport, guaranteeing superior Li‐storage capacity and high‐rate capability at high mass loading.^[^ ^9^, ^10^, ^11^ ^]^ However, the energy density of LIBs currently seems approaching the theoretical and practical limits. Also, hence, increasing attention and research are endeavored to active metal batteries by directly using Li, Na, K, Mg, Ca and Al, and Zn as anodes.^[^ ^12^, ^13^ ^]^ Unfortunately, they still face safety and limited energy/power performance issues from dendric growth, sluggish kinetics, low operating voltage, and material deactivation and loss.^[^ ^14^, ^15^, ^16^ ^]^

Anion intercalation into the active materials, as one of the most common types of insertion reactions, affording more positive working potential and higher energy density, is the cornerstone for designing dual‐ion batteries (DIBs).^[^ ^17^, ^18^ ^]^ Dai and co‐workers reported Al/pyrolytic graphite (PG) DIB by intercalating [AlCl_4_]^−^ into the cathode in the charge, exhibiting a superior high‐rate cyclability of ≈60 mAh g^−1^ at 3 kW kg^−1^ for 7500 cycles.^[^ ^19^ ^]^ Under reversible insertion/extraction of [AlCl_4_]^−^ in macrocycles, Choi and co‐workers designed an organic cathode DIB, delivering a high capacity of ≈95 mAh g^−1^ at 2 A g^−1^ and a retention of ≈59% after 5000 cycles.^[^ ^20^ ^]^ Intercalating various other anions such as tetrafluoroborate ([BF_4_]^−^), hexafluorophosphate ([PF_6_]^−^), and perchlorate ([ClO_4_]^−^) into graphite was also demonstrated at high voltages. Therefore, many graphite‐based DIBs have been developed as cost‐effective and high energy density candidates beyond LIBs. However, severe decomposition of electrolytes and increasing residue of anion radicals in the cathode at such higher voltage led to poor coulombic efficiency and fast degenerating of energy storage capability. Read et al. reported that the Li/graphite DIB with a high ceiling charge voltage up to 5.2 V delivered a capacity of ≈80 mAh g^−1^ for about ≈180 cycles with the coulombic efficiencies of ≈87–97%, but the capacity quickly faded afterward.^[^ ^21^ ^]^ Although the coulombic efficiency was improved to >95% without consideration of preliminary cycles by substituting the Li anode for a graphite electrode, unfortunately, the capacity still declined from ≈60 to ≈40 mAh g^−1^ after only 50 cycles.^[^ ^21^ ^]^ Also, hence, high concentration electrolytes and antioxidant additives are indispensable for most DIBs. Tang and co‐workers proved the low coulombic efficiency, and poor cyclability in Al/graphite DIBs could be significantly alleviated using 4 m LiPF_6_ in ethyl–methyl carbonate (EMC) electrolyte, along with 2% (wt%) vinylene carbonate (VC) additive to evolve stable solid electrolyte interphase (SEI) on the Al anode.^[^ ^22^ ^]^ The initial coulombic efficiency was elevated to ≈67%, and a high capacity of ≈104 mAh g^−1^ was achieved at 0.2 A g^−1^ along with 88% capacity retention for 200 cycles,^[^ ^22^ ^]^ whereas these properties are still far from satisfactory when comparing with LIBs. High‐rate preliminary activation processes involving many unknown complex chemical reactions are still essential to evolve a stable surface/interface protective layer to maintain an acceptable coulombic efficiency afterward and enable charge–discharge processes at a relative small rate feasible subsequently.^[^ ^21^ ^]^ Overall, the electrolytes in the DIBs are less exploited, and the coulombic efficiency, cycling stability, and energy/power performance are to be remarkably improved before practical application.^[^ ^18^, ^23^, ^24^ ^]^

**Scheme 1:**
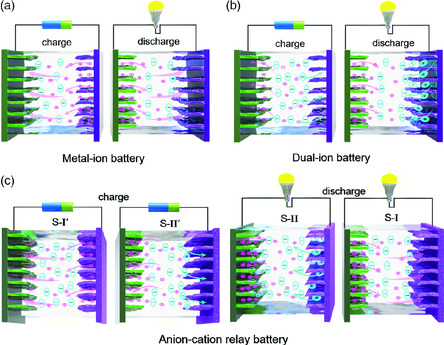
Illustration of the working mechanisms of three types of batteries. a) MIB. b) DIB. c) ACRB.

**Figure 1:**
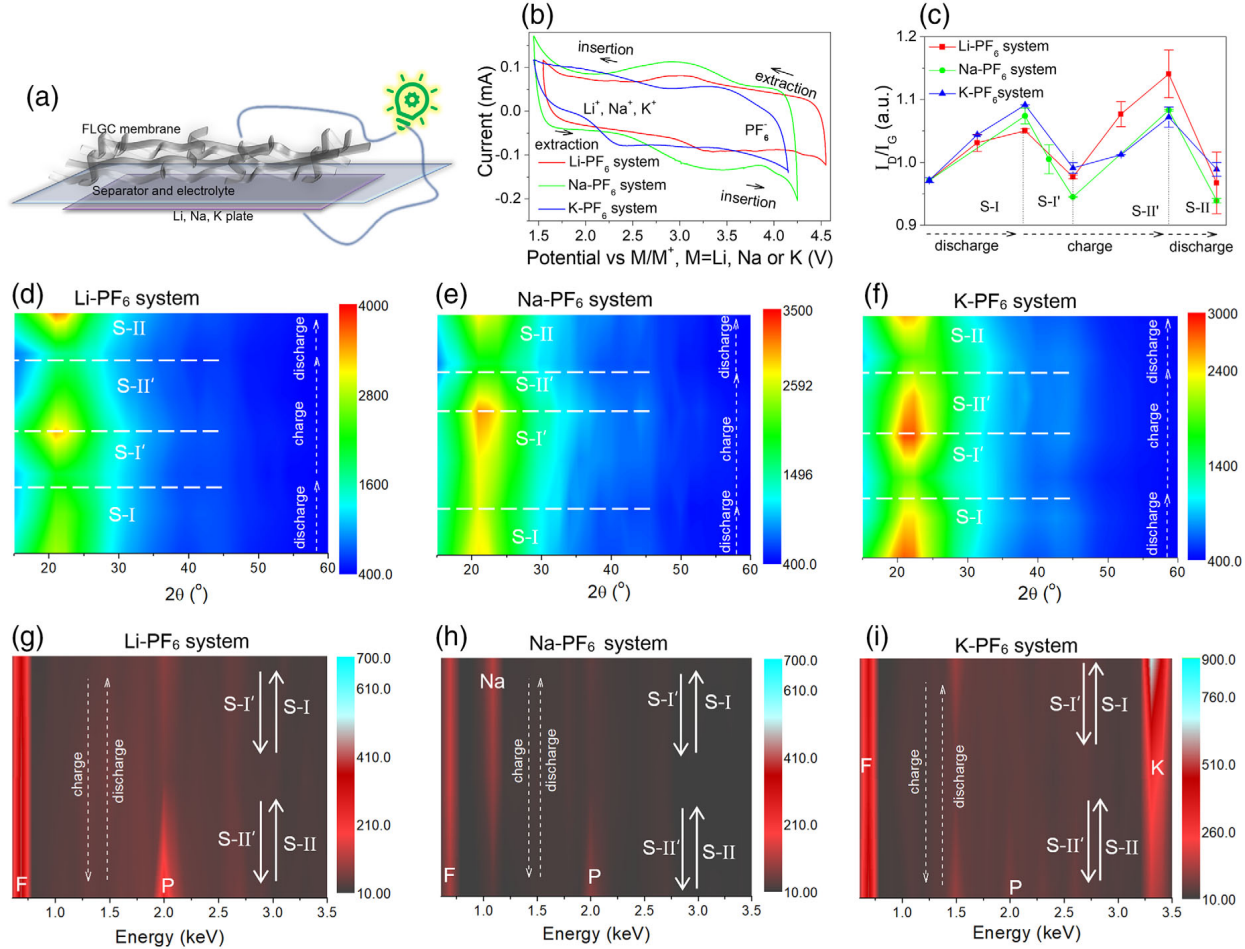
Analysis of the working mechanisms for three ACRB prototypes: a) battery assembly sketch, b) typical CV curves, and disorder–order change revealed by c) the variation of relative intensity (*I*_D_/*I*_G_) in Raman spectra, d–f) contour color map of powder XRD profiles, and relative element content change indicated by g–i) contour color map of EDX spectra of FLGC cathodes held at different SOCs.

Herein, we establish the basic concepts and clarify the working mechanism for the anion–cation relay batteries (ACRBs). In a traditional MIB **(** **Scheme** **1**a), the cathode suffers from insertion/extraction processes of positively charged metal ions in the discharge/charge processes, whereas similar opposite processes are performed by negatively charged anions in a DIB (Scheme 1b). Quite differently, two sequential stages of cation extraction (denoted as S‐I′) and anion insertion (denoted as S‐II′) happen in the new battery system during charging, whereas anion extraction (denoted as S‐II) and cation insertion proceed (denoted as S‐I) for the discharge reversely (Scheme 1c). We should note that the anode only performs cation insertion/extraction reactions, which can be intercalating/de‐intercalating, alloying/dealloying, or even plating/stripping, just like in the MIBs and DIBs.^[^ ^24^, ^25^, ^26^, ^27^ ^]^ In the proof‐of‐concept, we chose Li, Na, and K plates without any further surface/interface engineering as the anodes to simplify the processes of device assembly and mechanism analysis in the anode and simultaneously verify the reliability of the new energy storage model (see **Figure** **1**a for the battery assembly sketch), because only metal plating/stripping processes have occurred in these metal anodes, and we only need to clarify the reactions happening in the cathodes. ACRBs with tape‐cast few‐layered graphitic carbon (FLGC) electrodes as cathodes and Li, Na, and K plates as anodes fully verified the energy storage mechanism as expected (Li/FLGC cell with 1 m LiPF_6_ in ethylene carbonate (EC)/diethyl carbonate (DEC) (v/v = 1:1) electrolyte, Na/FLGC cell with 1 m NaPF_6_ in EC/dimethyl carbonate (DMC)/EMC (v/v/v = 1:1:1) and 5% fluoroethylene carbonate (FEC, wt%) electrolyte, and K/FLGC cell with 1 m KPF_6_ in EC/DMC/EMC (v/v/v = 1:1:1) and 2% FEC (wt%) electrolyte). Moreover, these ACRBs deliver impressive energy storage capability at various rates, excellent long‐term cycling stability, and high coulombic efficiency, outperforming similar counterparts of MIBs and DIBs and fully utilizing the electrolytes and promising to obtain more energy‐to‐cost ratios.

## 2 FLGC Membrane Cathode

To realize a breakthrough of coulombic efficiency and storage capability in the ACRBs, fast kinetics and abundant active sites in the cathodes should be ensured. We chose pyrolytic graphitic carbon from cellulose membrane as the cathode (see the digital optical photographs of FLGC membrane in Figure S1, Supporting Information). These FLGC membranes are characteristics of a 3D crosslinked architecture full of fibers, belts, and sheets of FLGC and mesopores as verified by the power X‐ray diffraction (XRD; Figure S2a, Supporting Information), energy dispersive X‐ray (EDX; Figure S2b, Supporting Information) spectrum, scanning electron microscopy (SEM; Figure S2c, Supporting Information), transmission electron microscopy (TEM; Figure S2d–f, Supporting Information), Raman spectrum, and nitrogen adsorption–desorption (Figure S3, Supporting Information) characterizations. The graphitic zones consist entirely of defect‐riched and edge‐oriented outward graphitic fragments of less than five layers and large interplanar spacing of ≈0.41 nm for (002) crystal planes. These features together guarantee smooth electron/ion transport and high efficiency and storage capability of insertion/extraction for both anions and cations.

## 3 Mechanism Analysis of FLGC Cathode in the ACRBs

In three ACRB systems, all the cyclic voltammogram (CV) curves scanned at 2 mV s^−1^ display at least more than one couple of anodic/cathodic peaks (Figure 1b) of almost the same covering area, implying similar reversible redox processes and faradic pseudocapacitive energy storage.^[^ ^9^ ^]^ Suppose, we can take them as the four stages in the ACRBs, and then, it seems that peak couples at the high voltage derive from insertion/extraction of [PF_6_]^−^, whereas the low voltage ones correspond to insertion/extraction of Li^+^/Na^+^/K^+^, respectively. It is to note that the terms “insertion/extraction” should include not only the intercalating/de‐intercalating process, but also not exclude surface/interface redox reaction from ions storage based on defects and O‐containing functional groups and other surface/interface adsorption/desorption processes, whereas the conclusion needs further convincing evidences. In the Raman spectra of graphites, D and G bands come from zone‐boundary phonons positively correlated with defect content and doubly degenerate zone center *E*_2g_ mode related to the sp2 bonding graphenic carbon layers, respectively.^[^ ^28^, ^29^ ^]^ Also, hence, the relative intensity of them (*I*_D_/*I*_G_) is applicable to analyze the defect content and size of graphenic zones in various graphitic carbon and graphenes. The sequential rise and decline in the statistical values of *I*_D_/*I*_G_ for the FLGC cathode at various stages of charge/discharge (SOCs; Figure 1c) confirmed a regular order–disorder change at the four stages of ACRBs, coinciding well with the predicable up–down or dilation–contraction of sp2 bonding carbon layers due to insertion/extraction of anions and cations.

Tape‐cast densification electrodes of FLGC were fabricated with a mixture of triturated free‐standing FLGC membrane, and polyvinylidene fluoride (PVDF) binder (FLGC/PVDF = 4:1 in wt%), so as to get a stronger signal in ex situ XRD characterization. The corresponding SEM images (Figure S4a,b, Supporting Information) affirm an enhanced compactness as expected. Evenly covered extra element of F on the carbon skeletons was observed in the element mapping images and EDX spectrum (Figure S4c–f, Supporting Information), indicating uniform distribution of the two components. Variation of XRD patterns of FLGC in ACRBs for the Li‐PF_6_ system held at different SOCs are shown in Figure S5, Supporting Information. However, we can hardly conclude the reasonable shift law of the reflections due to large peak width (>10°) because of poor crystalline character and very small crystalline zones in FLGC. Instead, the relative intensity of XRD reflections exhibits reasonable change due to ion insertion/extraction processes similar as the results of Raman. The contour color maps of the powder XRD profiles for the electrodes at different SOCs (Figure 1d–f) exhibit similar decreasing intensity in the reflections at ≈20° (2*θ*) corresponding to diffractions of (002) crystal planes of graphitic carbon when the FLGC electrodes undergo discharging stage of S‐I and charging stage of S‐II′, resulting from disordering of graphitic layers due to ion insertion. On the contrary, extraction of inserted ions out of the graphitic layers would lead to recovery of the peak intensity, quite consistent with the profile variation revealed at stages of S‐I′ during charging and S‐II during discharging. Actually, based on these above‐mentioned evidences, a clear sketch of the energy storage model revealing relay insertion/extraction of different ions can already be reasonably deduced.

**Figure 2:**
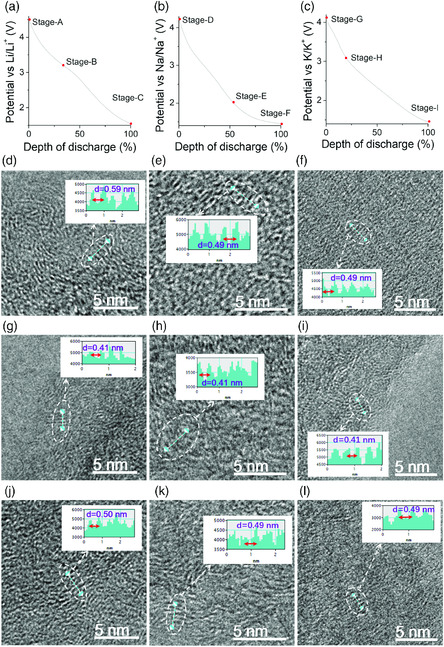
Analysis of the working mechanisms for three ACRB prototypes: a–c) typical discharge profiles with marked stages for FLGC cathodes held at different DODs and HRTEM images of FLGC at d) Stage‐A, e) Stage‐D, f) Stage‐G, g) Stage‐B, h) Stage‐E, i) Stage‐H, j) Stage‐C, k) Stage‐F, and l) Stage‐I.

To further ascertain these ions involved, element mapping images and EDX spectra for the FLGC electrodes at different SOCs (Figure S6–S8, Supporting Information, for the Li‐PF_6_ system, Figure S9–S14, Supporting Information, for the Na‐PF_6_ system, and Figure S15–S18, Supporting Information, for the K‐PF_6_ system) have been presented. Those mapping images depict obvious gathering of F element at the end of stage S‐II′ in the charge, and Na/K at the end of stage S‐I in the discharge, except for the absence of Li due to limitations of the EDX technique. The contour color maps of the EDX spectra at different SOCs (Figure 1g–i) reveal more direct evidence of the variation of F and P content in the charge/discharge stages of S‐II and S‐II′, verifying a reversible [PF_6_]^−^ insertion/extraction mechanism, whereas the intensity change of Na and K elements at the stages of S‐I and S‐I′ indirectly/directly attests the insertion/extraction reaction of Li, Na, and K cations. Moreover, prit surface at the discharge stage and granular surface at the charge stage for Li anodes both also indirectly substantiate the relay insertion/extraction processes in the ACRBs (Figure S19, Supporting Information). Except for the above‐mentioned macroscopic evidence of Raman, XRD, SEM, and EDX results, microscopic evidence from high resolution transmission electron microscopy (HRTEM) images at different depth of discharge stages (DODs) also provide us direct change of layer spacing of (002) crystal planes for the FLGC cathode due to a relay cation–anion insertion/extraction mechanism (**Figure** **2**). Combining all the evidences, we can clearly conclude that the insertion/extraction chemistry in the cathodes of the ACRBs involves reversible insertion/extraction reactions of both anions and cations in an alternate and sequential mode just like the scene in a relay race, accounting for the origin of the nomenclature of ACRB.

## 4 Electrochemical Performance for the Li‐PF_6_ Relay Batteries

**Figure 3:**
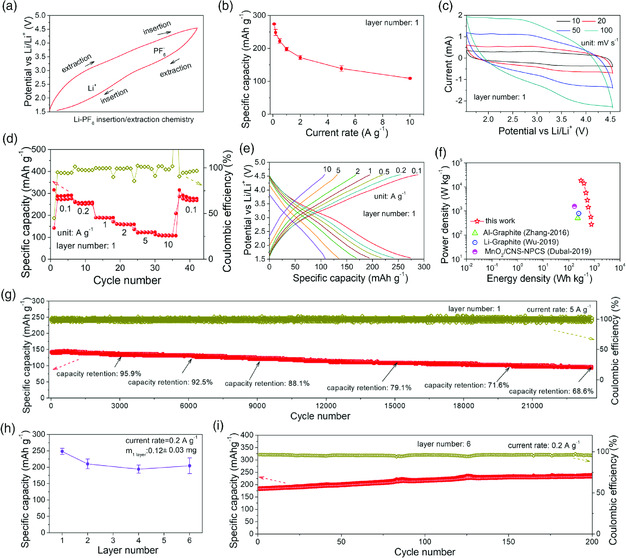
Electrochemical performance of Li‐PF_6_ relay battery: a) typical insertion/extraction chemistry in the cathode, b) capacity retention at different discharge rates, c) CV curves performed at different scan rates, d) rate performance and e) charge–discharge curves at various rates from 0.1 to 10 A g^−1^, f) Ragone plots with a comparison among this work and other common Li‐ion‐based energy storage devices, g) long‐term cycling stability tested at 5 A g^−1^, h) capacity retention, and i) cycling performance of the cathodes with different layers of membranes tested at 0.2 A g^−1^.

Specifically, the insertion/extraction chemistry in the FLGC cathode of Li/FLGC ACRB (**Figure** **3**a) can be outlined as that positively charged Li ions begin to extract from the lithiated FLGC in the initial charge stage at ≈2.2 V, and subsequently, anions of [PF_6_]^−^ start inserting into the graphitic layers at ≈3.8 V after the depletion of Li ions in‐taken previously, whereas reverse extraction/insertion of [PF_6_]^−^ anions and Li cations happen in the discharge. To in situ hold the fast electron/ion transport properties of pyrolytic graphitic carbon, free‐standing FLGC membrane of one single layer was directly used as the cathode. The corresponding ACRBs deliver the large capacities of 274, 248, 222, 197, 172, 138, and 109 mAh g^−1^ in a voltage window of 1.55–4.55 V at various discharge rates of 0.1, 0.2, 0.5, 1, 2, 5, and 10 A g^−1^, respectively (Figure 3b), twice more than those of DIBs.^[^ ^30^, ^31^ ^]^ The wide anodic/cathodic peaks in the CVs obtained at different rates (Figure 3c) indicate that faradic pseudocapacitance contributes a large proportion of the capacities at various rates, which also reveals a fast Li^+^/[PF_6_]^−^ insertion/extraction kinetics in the unique FLGC membrane (Figure S20, Supporting Information). In addition, a high insertion/extraction efficiency is also verified according to the good coulombic efficiencies of >94% at various rates (Figure 3d), without consideration of some sudden decline values due to rate alteration. The fast kinetics and high efficiency for the insertion/extraction reactions in the FLGC membrane finally evolve well‐defined charge–discharge slopes in the voltage profiles obtained at various rates (Figure 3e). The superior storage capability at various high rates also reflects an excellent energy/power performance of ≈722 Wh kg^−1^ at 561 W kg^−1^, ≈467 Wh kg^−1^ at 6.7 kW kg^−1^, and ≈300 Wh kg^−1^ at ≈18 kW kg^−1^ (Figure 3f), far outperforming the reported DIBs and cathodes of most advanced MIBs.^[^ ^22^, ^32^, ^33^ ^]^

Except for good energy storage capability and superior power performance, the Li/FLGC ACRB also demonstrates outstanding long‐term cycling stability exceeding 23 000 cycles with a high capacity retention of 68.6%, equivalent to a very tiny decay of ≈0.0013% per cycle (Figure 3g). Interestingly, the initial capacity of ≈140 mAh g^−1^ at 5 A g^−1^ persists in this decay ratio during the whole test, exhibiting a retention of ≈95.9% at 3000 cycles, ≈92.5% at 6000 cycles, ≈88.1% at 9000 cycles, ≈79.1% at 15 000 cycles, and ≈71.6% at 20 000 cycles. Also, hence, the regular decline of capacity can be reasonably ascribed to gradual degeneration Li‐plating/‐stripping processes in the undesirable Li plate, which readily lead to slight deviation in actual internal voltage.^[^ ^13^ ^]^ More importantly, the storage capability and cycling stability are weakly affected by the membrane layers (Figure 3h), and the cell with six layers of free‐standing FLGC membrane still delivers an average storage capacity of ≈202 mAh g^−1^, which further rises to ≈231 mAh g^−1^ in 200 cycles at 0.2 A g^−1^ probably due to the penetration hysteresis of electrolyte between layers (Figure 3i), showing very little discount of less than 7% comparing with that of a single‐layer cell.

## 5 Electrochemical Performance for the Na‐PF_6_ Relay Battery

**Figure 4:**
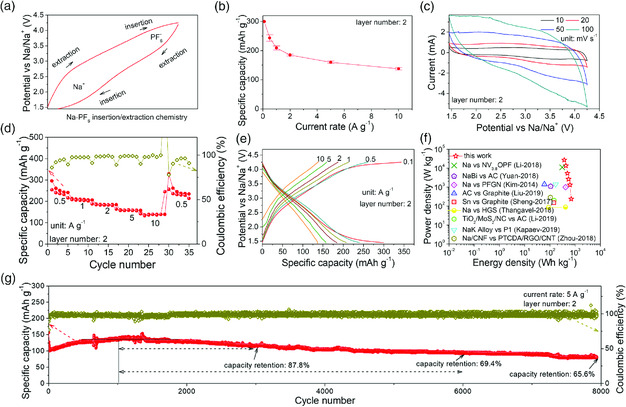
Electrochemical performance of Na‐PF_6_ relay battery: a) typical insertion/extraction chemistry in the cathode, b) capacity retention at different discharge rates, c) CV curves performed at different scan rates, d) rate performance at various rates from 0.5 to 10 A g^−1^, e) charge–discharge curves at various rates from 0.1 to 10 A g^−1^, f) Ragone plots with a comparison among this work and other common Na‐ion‐based energy storage devices, and g) long‐term cycling stability tested at 5 A g^−1^.

The Na/FLGC Na‐PF_6_ relay battery also follows the alternate insertion/extraction chemistry of anions and cations. As shown in the typical voltage profiles (**Figure** **4**a), insertion/extraction of Na ions happens at ≈2/2.5 V, respectively, whereas at ≈3.5/3.8 V do the insertion/extraction of [PF_6_]^−^ occur. The corresponding cell with two layers of FLGC membrane as the cathode also exhibits an impressive storage capability of ≈300 mAh g^−1^ at 0.1 A g^−1^ as expected (Figure 4b). Anodic/cathodic peaks in the CVs performed at various sweep rates (Figure 4c) substantiate similar considerable contribution of faradic pseudocapacitance (Figure S21, Supporting Information). The rate performance tested in a window voltage of 1.45–4.25 V (Figure 4d) shows that the high capacities of ≈254, 210, 185, 160, and 138 mAh g^−1^ are achieved at the sequential varying rates of 0.5, 1, 2, 5, and 10 A g^−1^, respectively. A full recovery capacity of ≈255 mAh g^−1^ has been retained after the rate backs to 0.5 A g^−1^. Charge–discharge slopes attributed to insertion/extraction of anions and cations are also discernable in the voltage profiles performed at various current rates (Figure 4e). The corresponding Ragone plots (Figure 4f) reveal that a superior energy density of ≈700 Wh kg^−1^ is held at a power density of ≈240 W kg^−1^; meanwhile, a high capacity of ≈138 mAh g^−1^ is achieved in a short charge–discharge duration of 49 s, amounting to an unparalleled energy/power performance of ≈370 Wh kg^−1^ at ≈27 kW kg^−1^ far ahead of Na‐based DIBs and Na‐ion‐based advanced cathodes.^[^ ^34^, ^35^, ^36^, ^37^, ^38^, ^39^, ^40^, ^41^ ^]^ Moreover, long‐term galvanostatic charge–discharge test carried at 5 A g^−1^ (Figure 4g) also proves the stable cycling performance, holding a high capacity retention of 65.6% for nearly 8000 cycles just corresponding to an average decline of ≈0.0043% for each cycle.

## 6 Electrochemical Performance for the K‐PF_6_ Relay Battery

**Figure 5:**
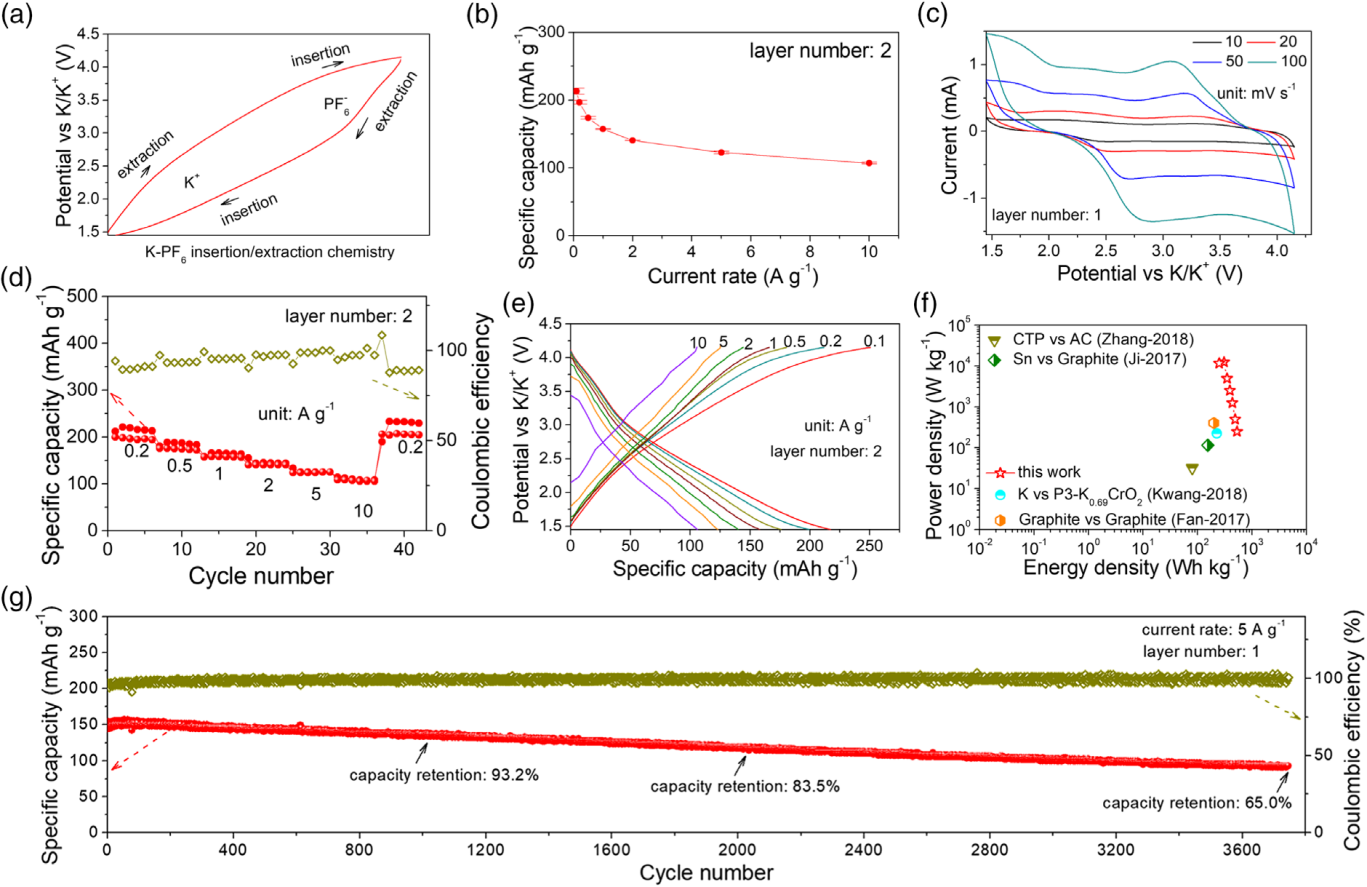
Electrochemical performance of K‐PF_6_ relay battery: a) typical insertion/extraction chemistry in the cathode, b) capacity retention at different discharge rates, c) CV curves performed at different scan rates, d) rate performance at various rates from 0.2 to 10 A g^−1^, e) charge–discharge curves at various rates from 0.1 to 10 A g^−1^, f) Ragone plots with a comparison among this work and other common K‐ion‐based energy storage devices, and g) long‐term cycling stability tested at 5 A g^−1^.

Comparing with the above‐mentioned two cells, the K‐PF_6_ relay battery exhibits relatively slower slopes for K insertion/extraction at ≈1.7/2.7 V in the voltage profile (**Figure** **5**a), appearing marked shifts due to increasing barriers as a result of enlarged ionic radius and heavier atomic mass (relative atomic mass (a. u.): K ≈39, Na ≈23, Li ≈7 and ionic radius (nm): K^+^ ≈0.138, Na^+^ ≈0.102, Li^+^ ≈0.076). The variation also gives rise to an obvious capacity shrinkage of ≈80 mAh g^−1^ (≈217 mAh g^−1^ at 0.1 A g^−1^ in Figure 5b) for the K‐PF_6_ relay battery with two layers of free‐standing FLGC membrane. Evidenced by the invariable insertion/extraction potentials of ≈3.5/3.8 V, the intercalating of [PF_6_]^−^ was weakly affected. The severe hysteresis is also observed in the CVs performed at different sweep rates **(**Figure 5c). Actually, for such large radii, diffusion‐controlled energy storage will also be extremely depressed, as reflected by the increasing capacitive contribution ratio with the ionic radius ascending in the sweep voltammetry analysis (Figure S20–S22, Supporting Information). Interestingly, as the sweep rates rise, increasing capacitive contribution ratio in various MIBs is not applicable to the ACRBs, which exhibit enhanced diffusion‐controlled energy storage due to stronger power to drive more ions insertion/extraction at higher rates.^[^ ^40^ ^]^ Even so, a superior rate performance of ≈200, 175, 157, 141, 124, and 110 mAh g^−1^ is still obtained at the varying rates from 0.2 to 10 A g^−1^ with a voltage window of 1.45–4.15 V (Figure 5d,e). The good high‐rate performance endows the cell with a high energy density of ≈305 Wh kg^−1^ at large power density of ≈12 kW kg^−1^ (Figure 5f) much better than those of K‐based DIBs and K^+^‐based cathodes of MIBs.^[^ ^42^, ^43^, ^44^, ^45^ ^]^ As the cells are galvanostatically tested at 5 A g^−1^, a similar stable cycling performance with a high coulombic efficiency of ≈97.5 ± 3.8% is maintained for ≈3700 cycles with a slight retention decay of ≈0.009% per cycle. Although almost all of the coulombic efficiencies are higher than 90%, it is to note that the slight drop in coulombic efficiency and a remarkable decrease in lifespan of Na/K‐based ACRBs are attributed to the relatively more unstable plating/stripping processes in Na/K plates anode in contrast to Li.^[^ ^36^, ^42^, ^45^ ^]^

## 7 Prospects of the ACRBs

When we focus on the ACRBs, our choice of electrodes should not be limited to graphite and Li/Na/K. For the proposed ACRB prototypes in this manuscript, the cycling stability could be further improved using various more stable active metal nanoarchitecture anodes reported previously.^[^ ^8^, ^13^ ^]^ Except for plating/stripping, alloying/dealloying and intercalating/de‐intercalating can also be introduced to strengthen the stability, storage capability, and compactness of anodes in the electrode design.^[^ ^24^, ^25^, ^46^, ^47^ ^]^ Meanwhile, various current advanced cathodes of phosphates, layered oxides and sulfides, fluorides, vanadates and manganates, etc. can be coupled with graphite and graphitic derivatives for significant breakthrough in the high energy density of cathodes, along with desirable power performance. If carbon nanostructures or Si that without a sources of metal ions were chosen as the anode, the cathodes should function as metal ion sources, e.g., LiFePO_4_/graphite hybrid nanostructures. In this case, the cell is in discharged state, and it should be first charged, with Li^+^ extraction from LiFePO_4_ and then the anions intercalating into graphite or interfaces. If advanced nanostructures of Li metal or Li alloy were the anode, then the cathode could be metal fluorides/graphite, metal oxides/graphite, or other similar hybrid composites without the sources of metal ion. In this case, the cell is in a charged state, and it can be discharged first as the described ACRB in the manuscript. All in all, the concept for the ACRB is well compatible with the chargeable battery systems of MIBs and DIBs.

## 8 Conclusion

In summary, we have proposed a new energy‐storage paradigm for non‐aqueous rechargeable batteries. Cells based on the paradigm fully utilize both the cations and anions by alternate and sequential insertion/extraction reactions at the cathodes. With the aid of Raman, XRD, SEM, TEM, and EDX characterization techniques, we have validated that three ACRB prototypes just worked as expected. Benefiting from full exploitation of both anions and cations, three ACRBs with tailored free‐standing FLGC membrane cathodes exhibit excellent energy storage capacities at various rates and high coulombic efficiency and stable long‐term cycling performance, exceeding most DIBs and MIBs. We rationally speculate that the energy density of the ACRBs will double that of DIBs and outperform the LIBs by at least 20% increase with properly tailored anodes and cathodes in the future.

## 9 Experimental Section

9.1

9.1.1

##### Material Preparation and Characterization

Free‐standing FLGC membrane was obtained directly by pyrolytical treatment of ultrathin cellulose membrane delaminated from roll paper at 1000 °C for 6 h in a tube furnace at vacuum atmosphere. Trituration of FLGC was performed in a degassed polyethylene (PE) bag. Microstructure and morphology of FLGC membrane and FLGC electrodes were characterized by thermal field emission SEM (Quanta 400F, 20 kV) and field emission TEM (FEI Tecnai G2 F30, 300 kV). Structure and elemental analysis were performed by XRD (D/MAX 2200, Cu kα1, 5° min^−1^), EDX spectrum and element mapping, and Raman (Renishaw, 514.5 laser).

##### Ex Situ Characterization of FLGC Electrodes

For a mechanism analysis, a slurry mixture containing FLGC and PVDF (FLGC/PVDF = 4:1 in wt%), and 1‐methyl‐2‐pyrrolidinone (NMP) as the dispersion solvent was made. The slurry mixture was casting on Al foil by an automatic thick film coater. Then, the coating film was desiccated in a vacuum chamber at 90 ^°^C for 12 h. After that the foil is pressed by an electromotive roller and tailored to appropriate size by a coin‐type cell microtome. The FLGC layer peeled off the Al foil was used as the cathode (≈5–10 mg for each piece) for mechanism characterization. The cathodes from different disassembled cells were immersed in DEC for 6 h to wash off the electrolyte (DMC for Na‐based and K‐based ACRBs), and then held for ≈5 days for natural drying in an Ar‐filled glove box. These dry electrodes were directly used for ex situ XRD, SEM, and Raman characterization.

##### Battery Assembly and Measurement

For electrochemical tests, free‐standing FLGC membrane of different layers was tailored to an appropriate size (*ϕ* = 1.2 cm, ≈0.12 ± 0.03 mg for each layer) and directly used as the cathodes, and Li, Na, and K plates in the same size were used as the anodes for Li/FLGC, Na/FLGC, and K/FLGC cells, respectively. The corresponding electrolytes were 1 m LiPF_6_ in EC/DEC (v/v = 1:1), 1 m NaPF_6_ in EC/DMC/EMC (v/v/v = 1:1:1) and 5% FEC (wt%), and 1 m KPF_6_ in EC/DMC/EMC (v/v/v = 1:1:1) and 2% FEC (wt%), respectively. Standard cells (CR2032) with the tailored electrodes mentioned earlier, polypropylene micromembrane (Celgard 2500) separator, and 25 μL electrolyte were assembled in an Ar‐filled universal glove box with the oxygen and water vapor pressure less than 0.3 ppm.

The cycling and rate performance were galvanostatically tested in a voltage cutoff of 1.55–4.55 V for the Li‐PF_6_ system, 1.45–4.25 V for the Na‐PF_6_ system, and 1.45–4.15 V for the K‐PF_6_ system at various rates on a multichannel Neware battery testing system. CVs at the same cutoff voltage and different sweep rates from 1 to 100 mV s^−1^ were carried out on an Ivium electrochemical workstation. Cells for mechanism analysis were galvanostatically discharged and/or charged to different cutoff voltages at 50 mA g^−1^.

## Conflict of Interest

The authors declare no conflict of interest.

## Supporting information

Supplementary Material
