# Supplementary material for: The Anion–Cation Relay Battery Prototype
Source: Small Sci. 2020 Nov 12;1(1):2000030. doi: 10.1002/smsc.202000030 (PMC11936006; doi:10.1002/smsc.202000030)
Supplement: Supplementary file 1 — Supplementary Material [file SMSC-1-2000030-s001.docx]

The Anion-Cation Relay Battery Prototype

**Huawei Song,^1*^ Jian Su,^1^ Chengxin Wang^1*^**

^1^State Key Laboratory of Optoelectronic Materials and Technologies, School of Materials Science and Engineering, Sun Yat-Sen (Zhongshan) University, Guangzhou 510275, People’s Republic of China E-mail: songhw5@mail.sysu.edu.cn; wchengx@mail.sysu.edu.cn


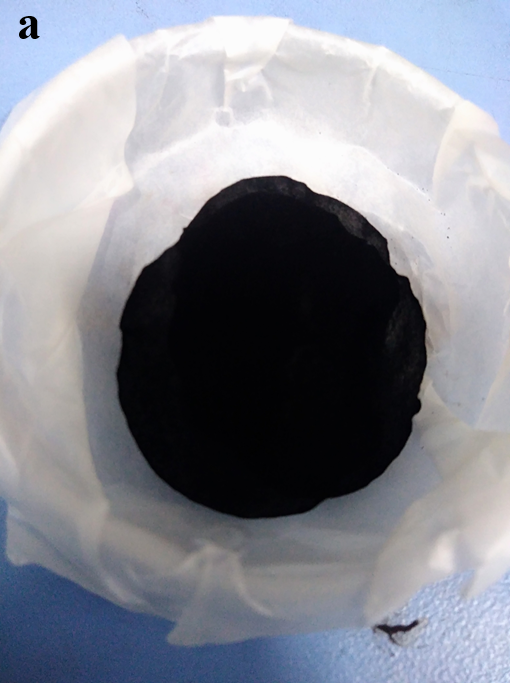

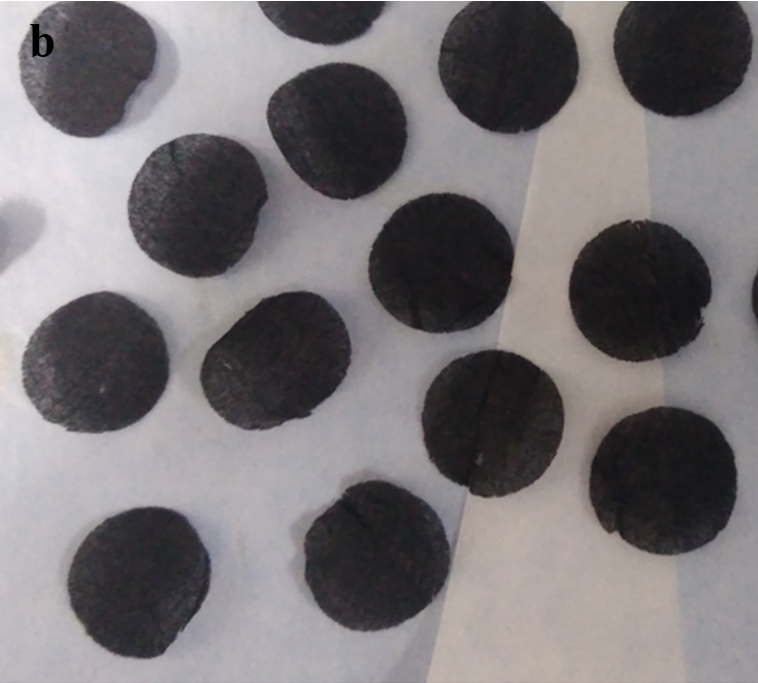


**Figure S1.** **Digital optical photographs of the free-standing FLGC membrane.** **a**) Stacking four layers with a diameter of 3.5 centimeters, **b**) tailored single layer electrode with a diameter of 1.2 centimeters.


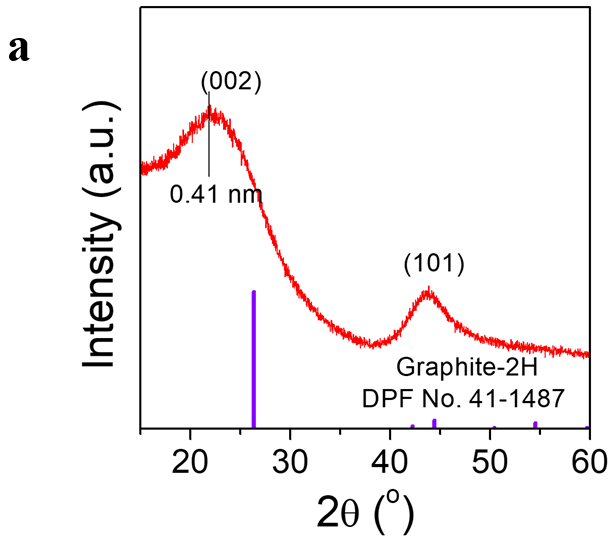

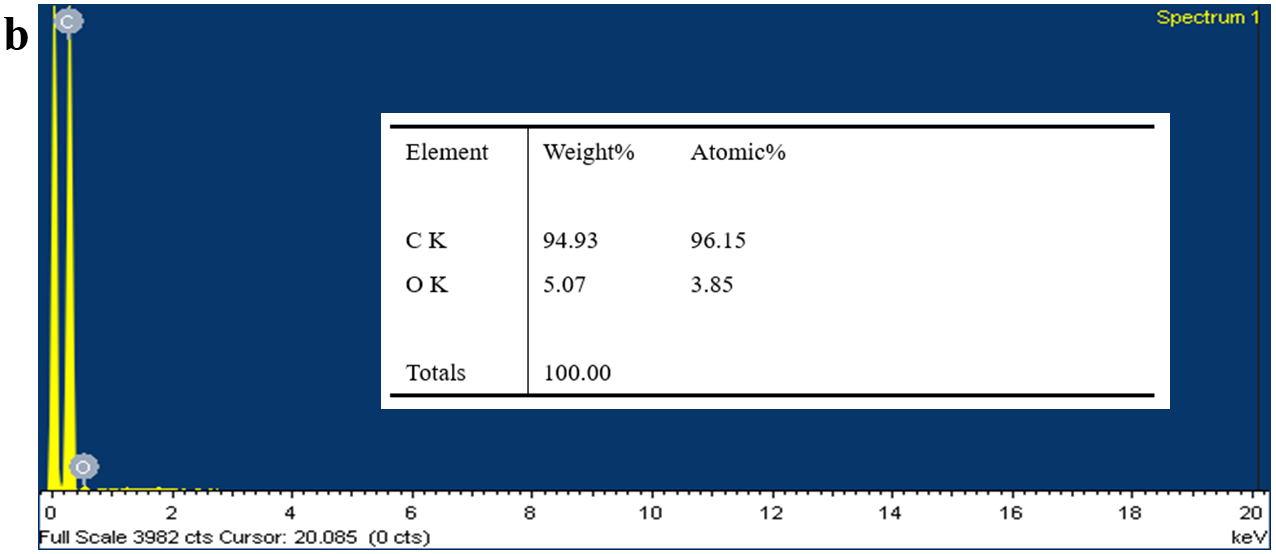

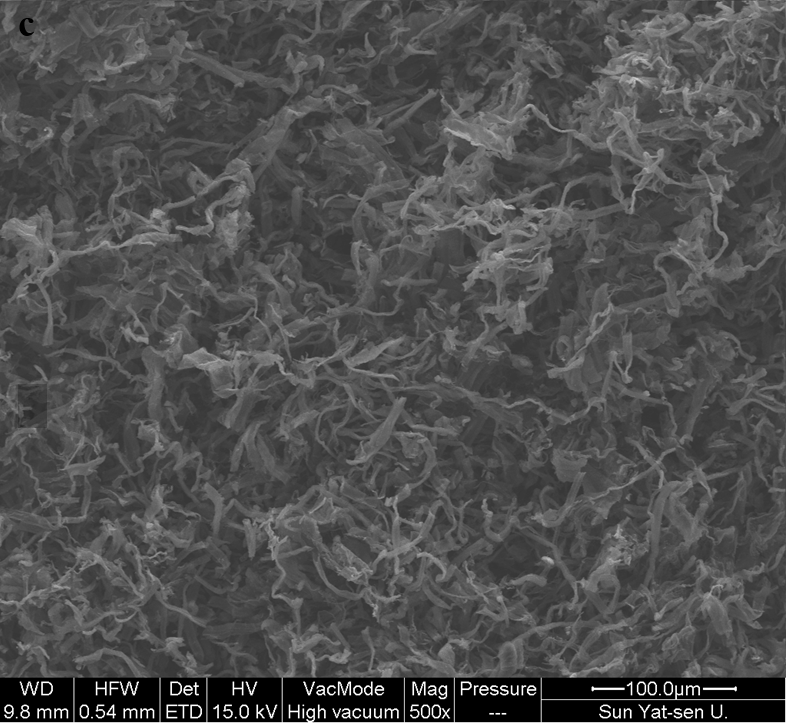

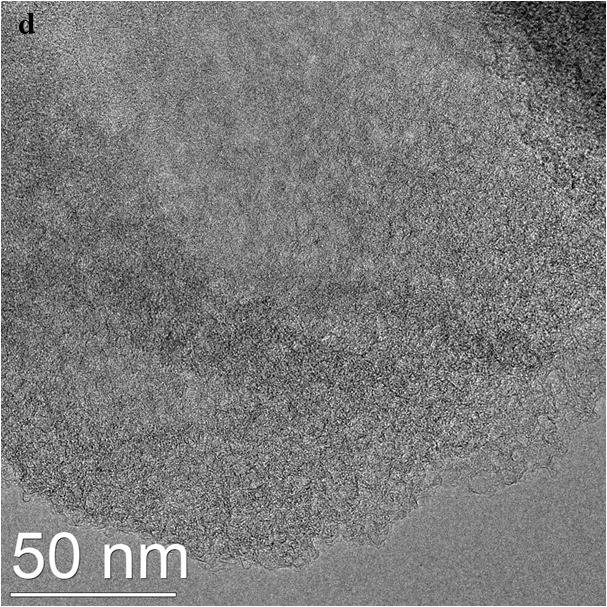

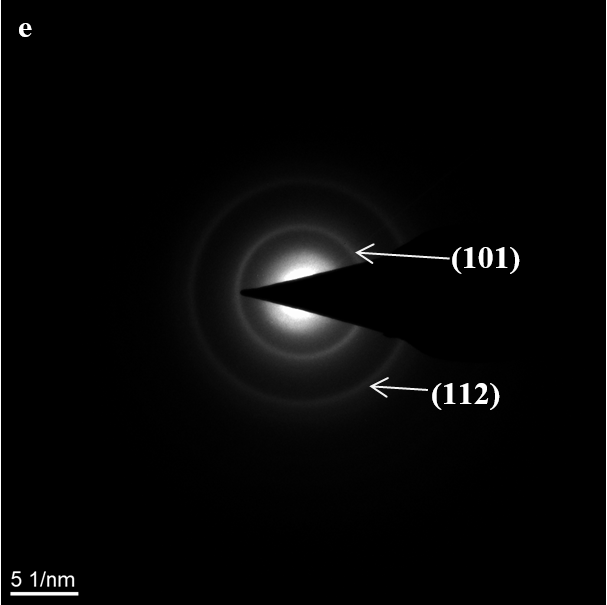

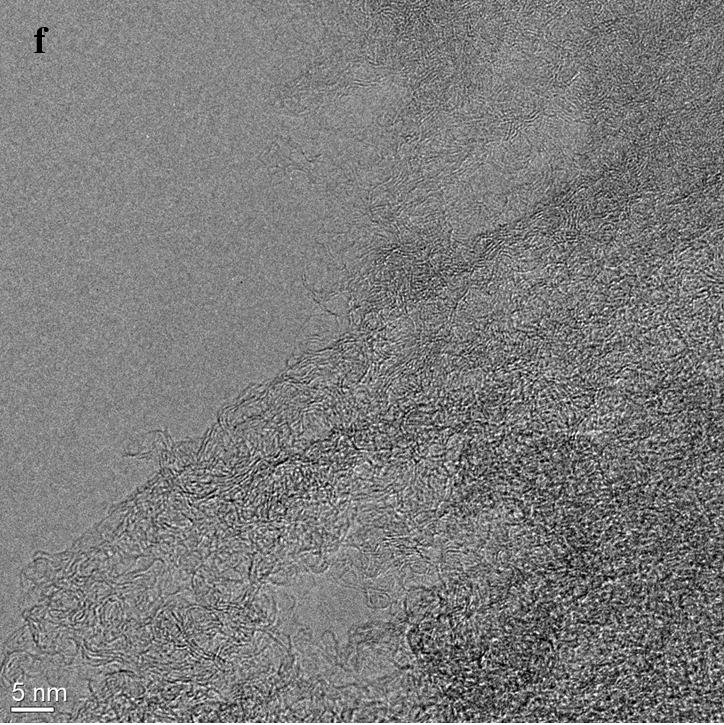


**Figure S2**. **Phase, composition, morphology, and microstructure of the as-prepared FLGC membrane.** **a**) Powder XRD pattern in comparison with that of standard graphite, **b**) EDX spectrum and the element content, **c**) Low magnification SEM image showing the flexible fiber-like or tangled-sheet-like units, **d**) TEM image presenting the porous microstructure in each unit and **e**) corresponding SAED image, **f**) HRTEM image indicating the abundant defect-riched few-layered graphitic components and edge-oriented outward graphitic fragments.


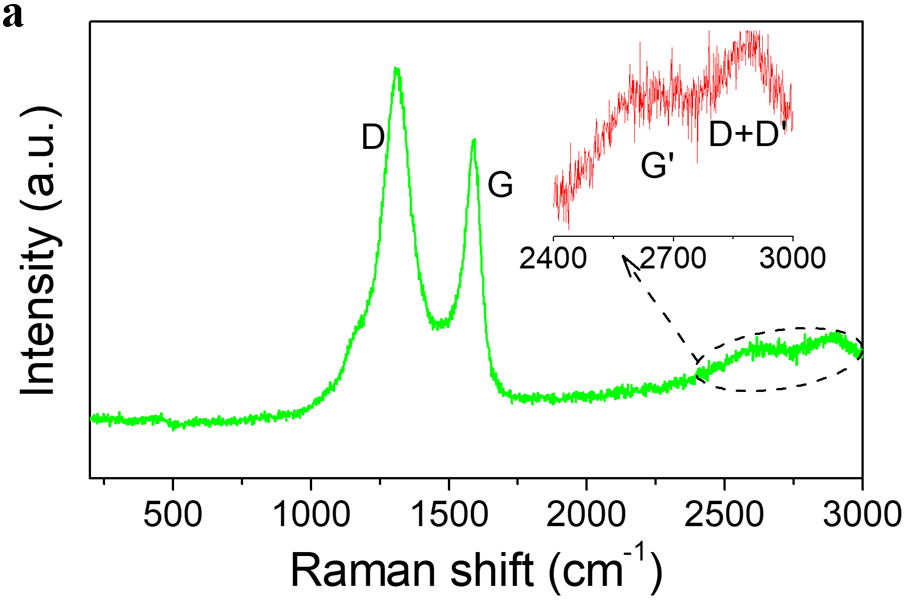

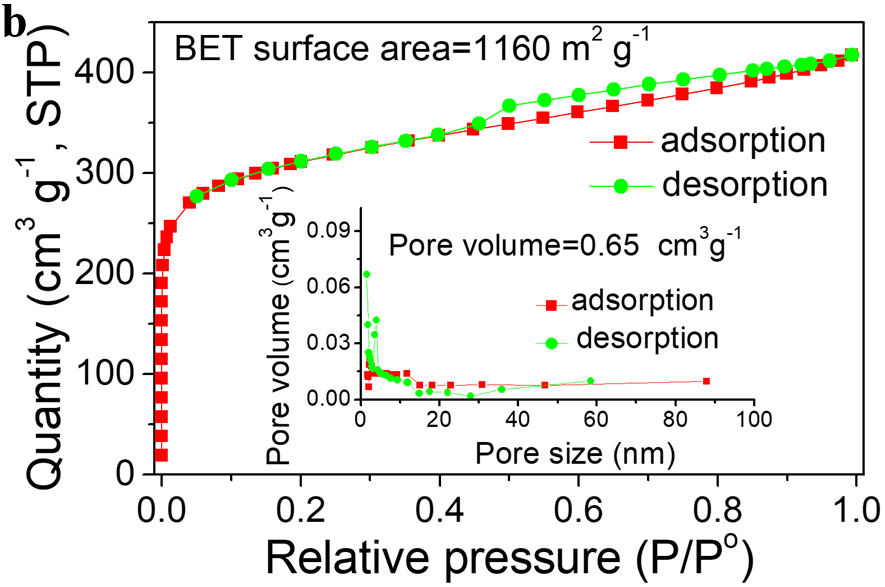


**Figure S3.** **a**) Raman spectrum and **b**) surface and pore analysis for FLGC membrane.


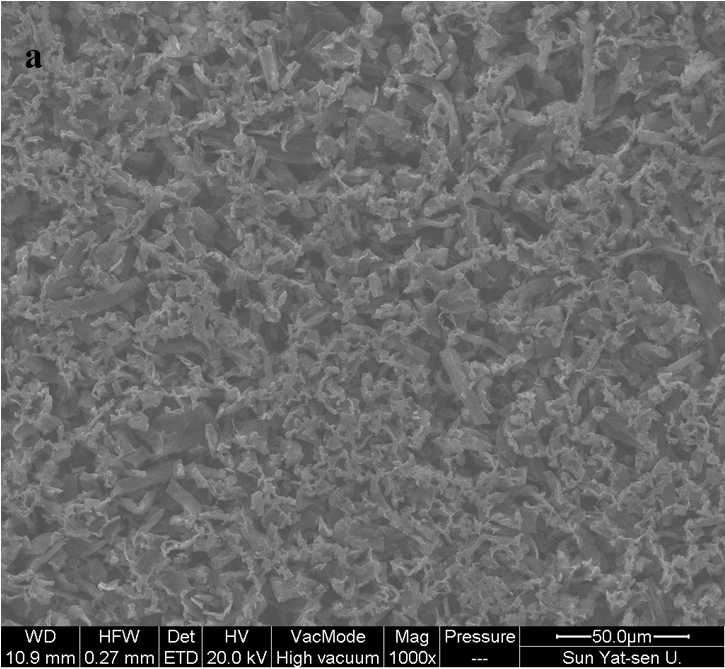

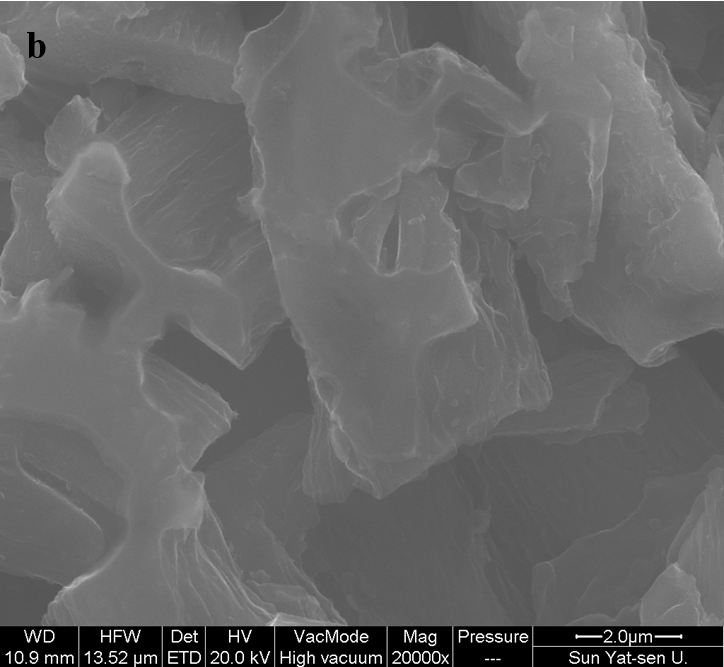


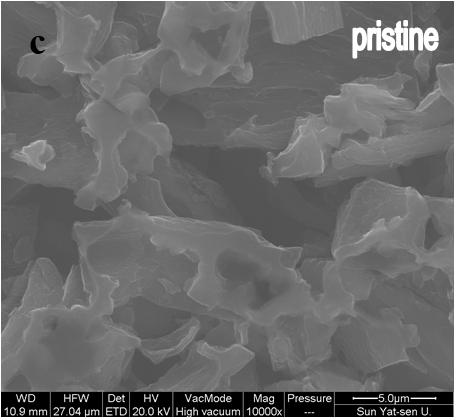

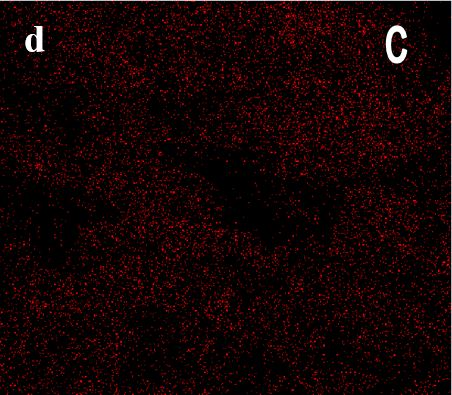

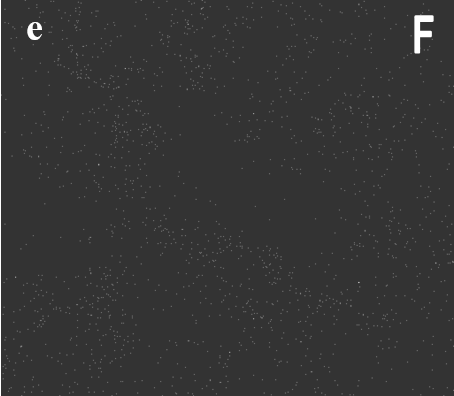


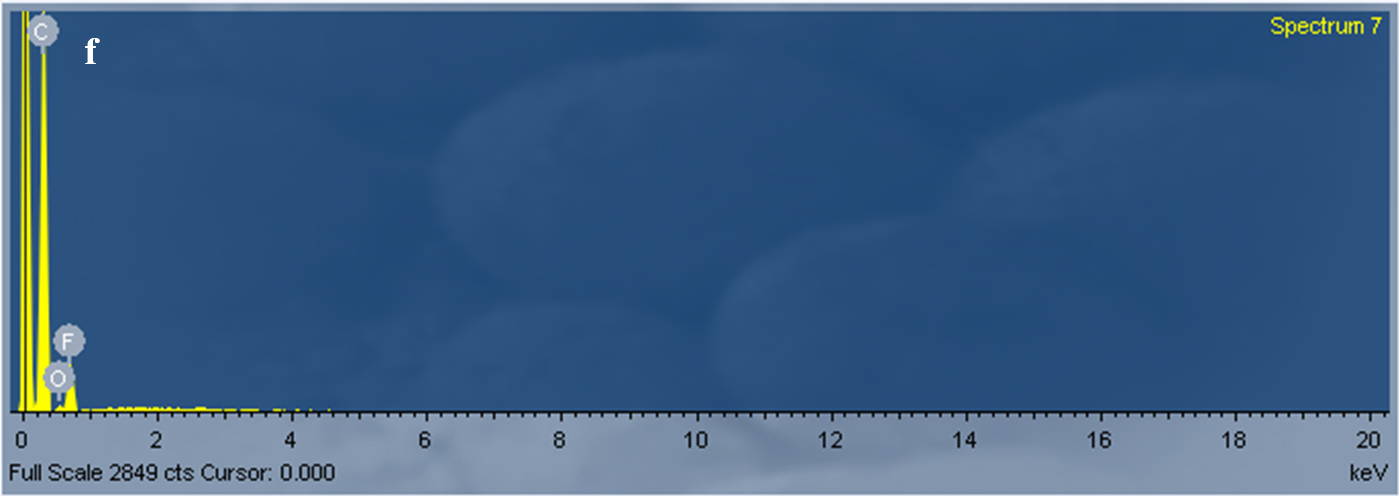


**Figure S4.** **Tape-cast electrode of FLGC in high compactness for mechanism characterization, the weight ratio of FLGC to PVDF is controlled to 4:1, and PVDF is a common binder used in battery fabrication.** **a-b**) SEM images, **c-e**) element mapping images, **f**) the corresponding EDX spectrum.


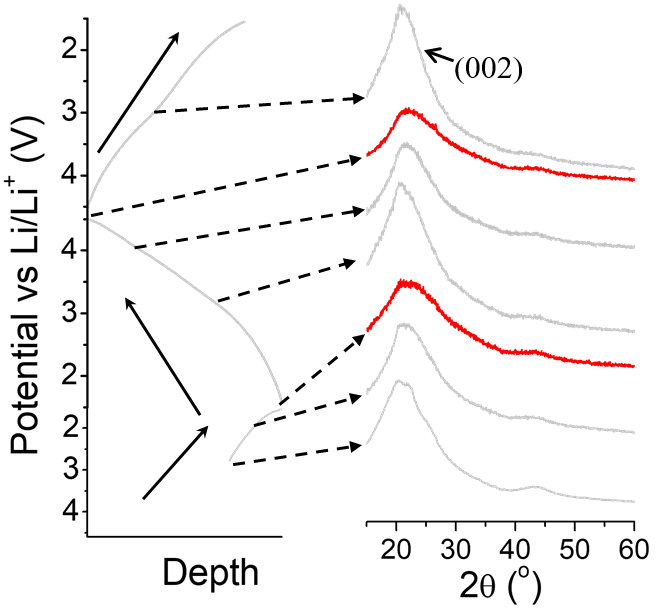


**Figure S5.** Variation of XRD profiles of FLGC cathodes in Li-PF_6_ ACRBs held at different stage of charge/discharges (SOCs).


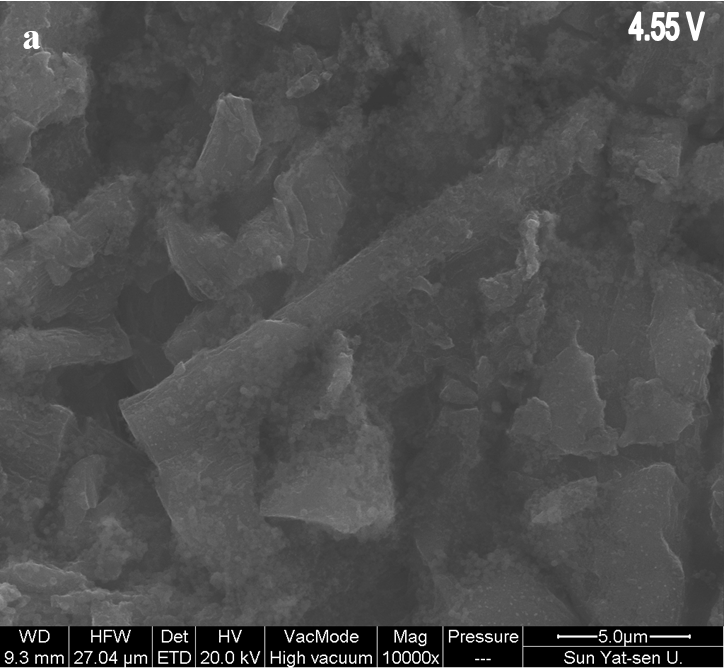

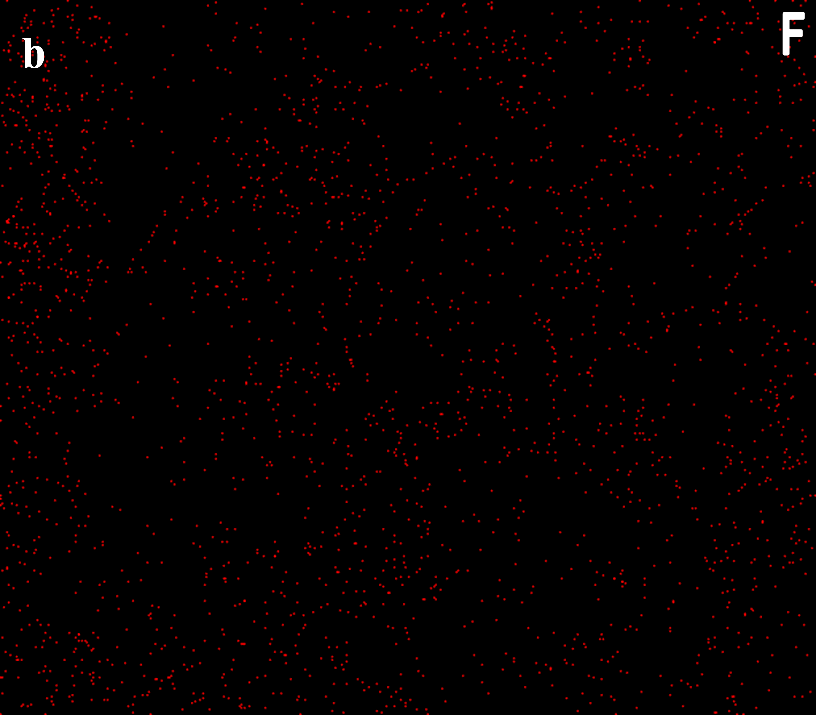

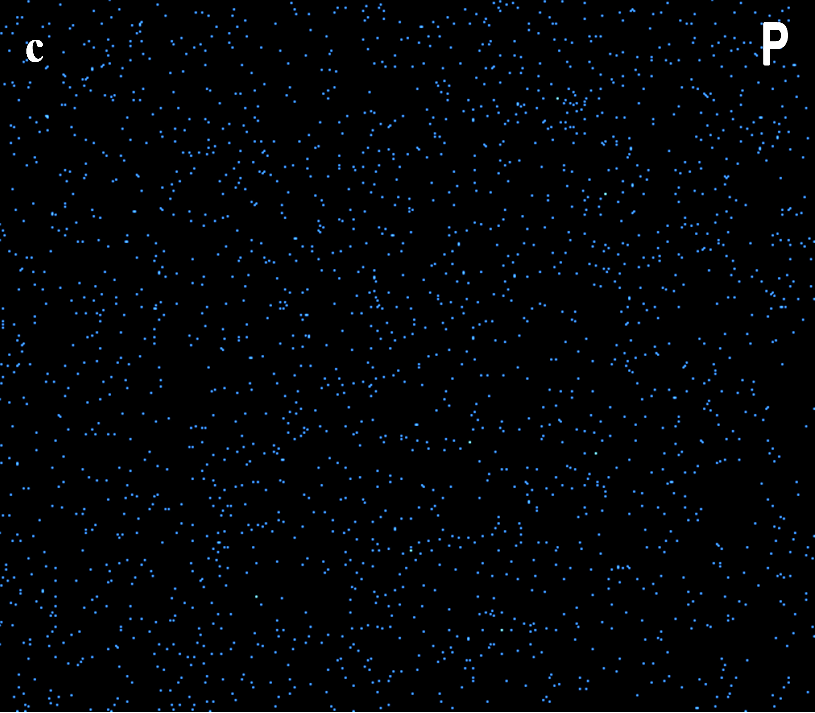

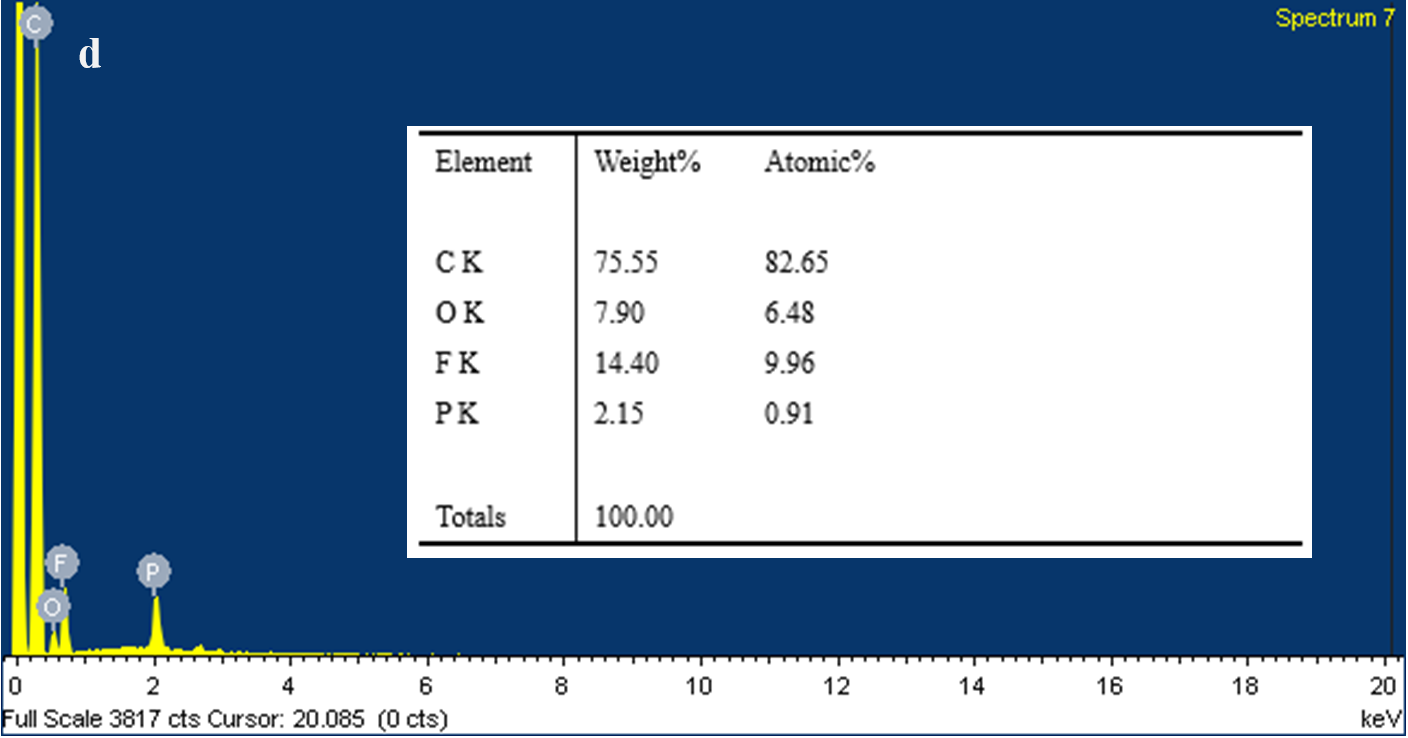


**Figure S6.** **Element mapping images and EDX spectrum for FLGC tape-cast electrode held at a charge stage of 4.55 V in the Li-PF_6_ relay battery. a-c**) SEM and element mapping images, **d**) the corresponding EDX spectrum.


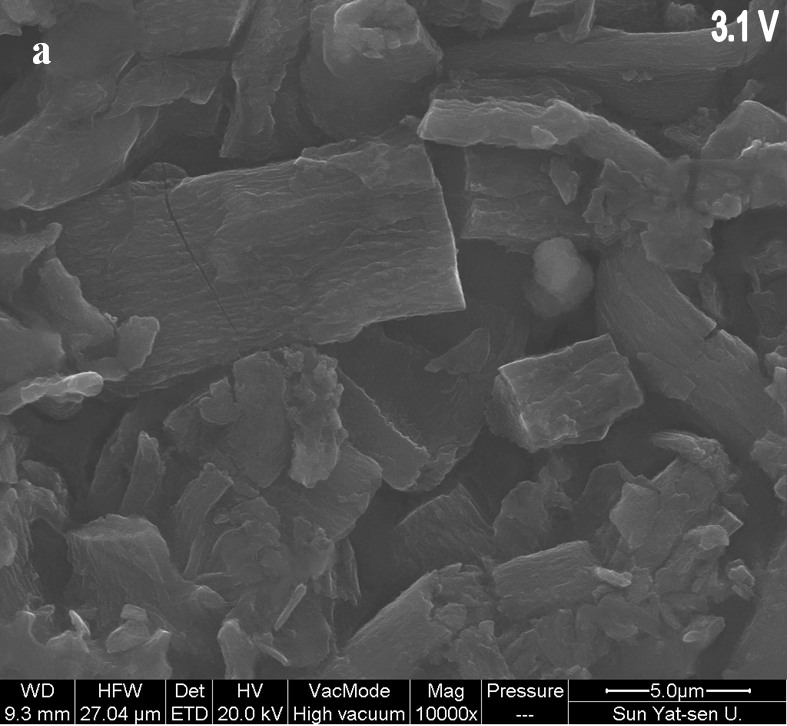

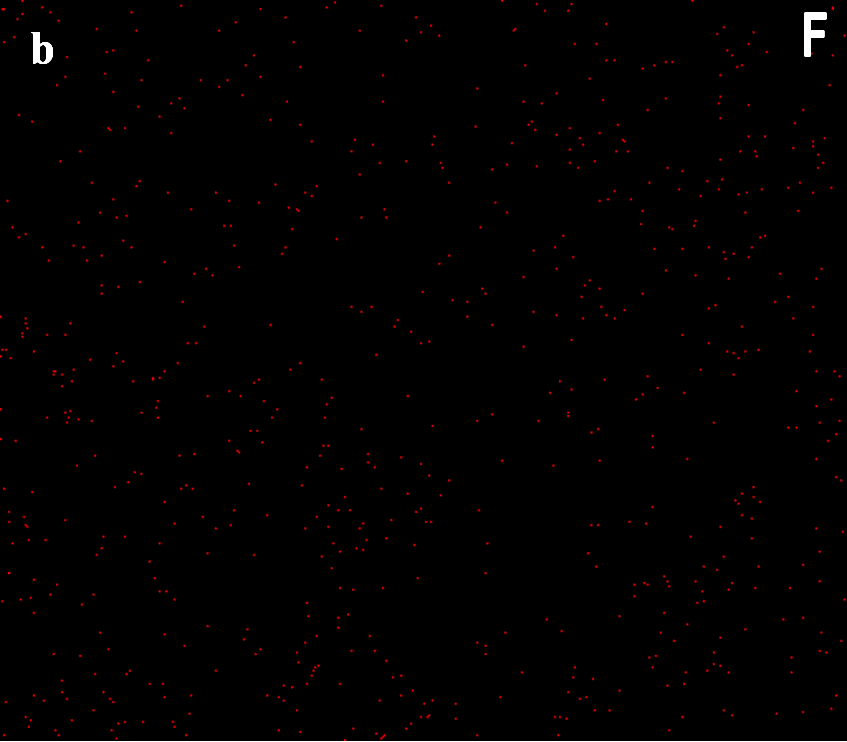

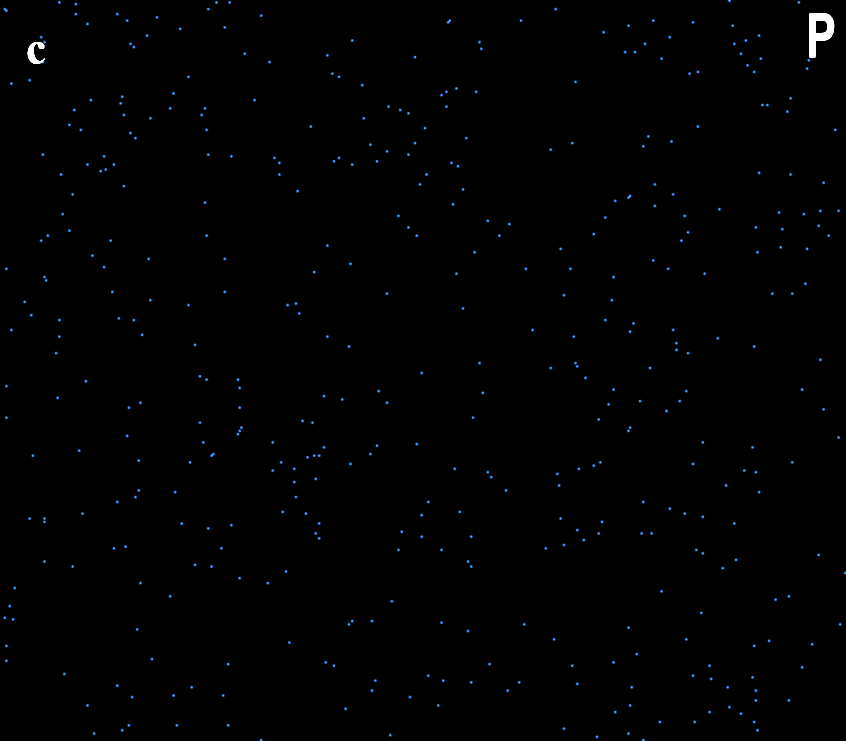

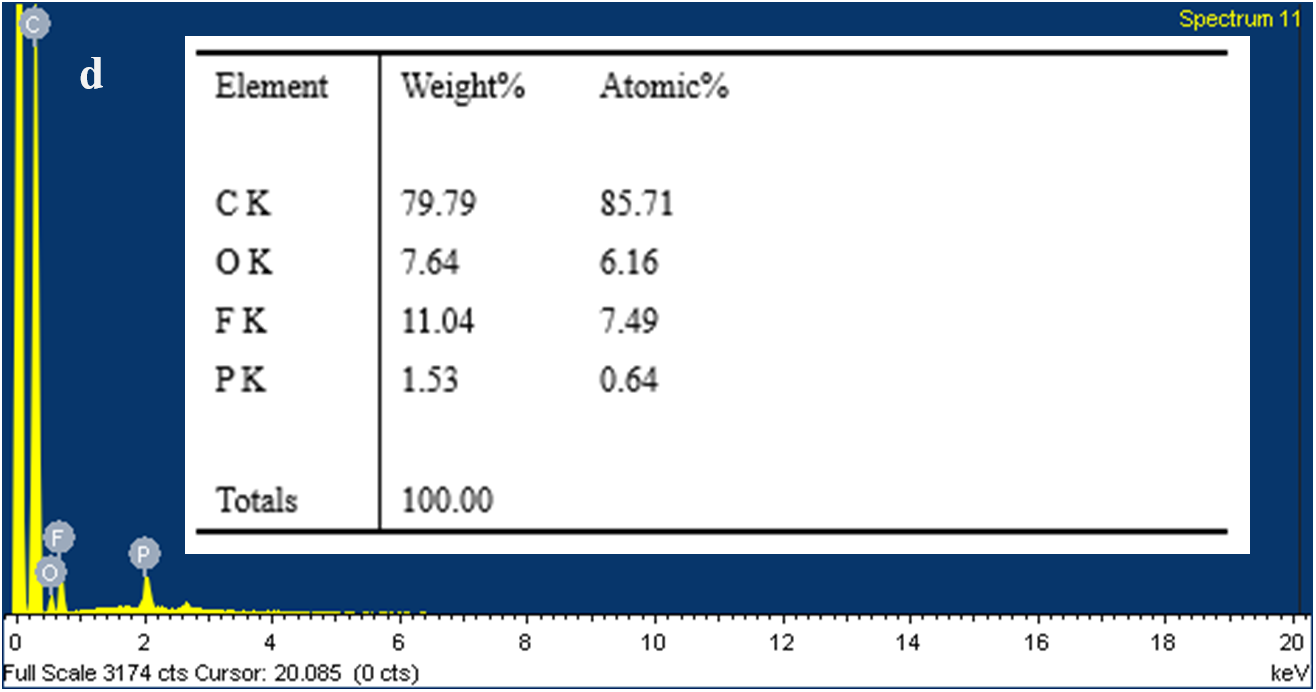


**Figure S7.** **Element mapping images and EDX spectrum for FLGC tape-cast electrode held at a discharge stage of 3.1 V in the Li-PF_6_ relay battery.** **a-c**) SEM and element mapping images, **d**) the corresponding EDX spectrum.


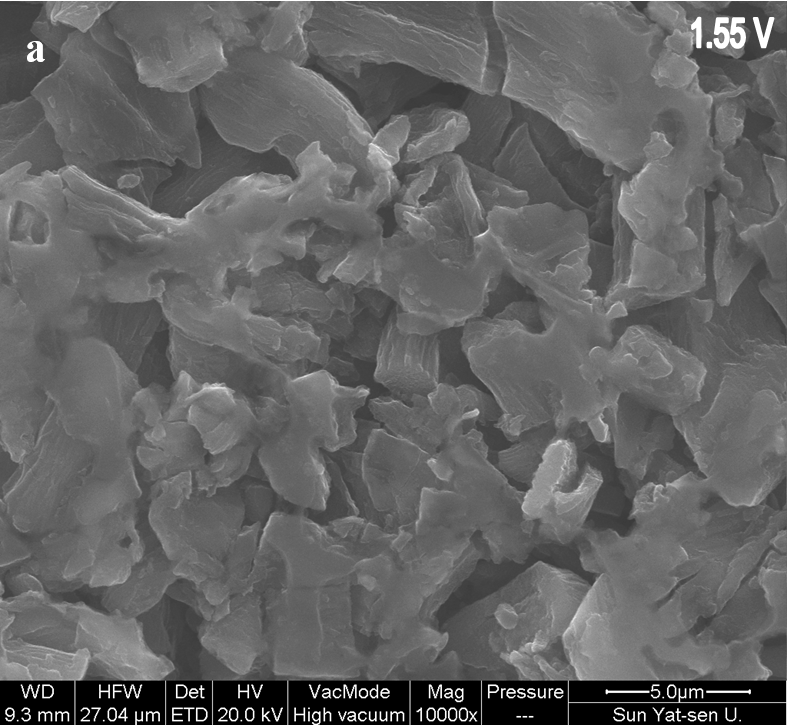

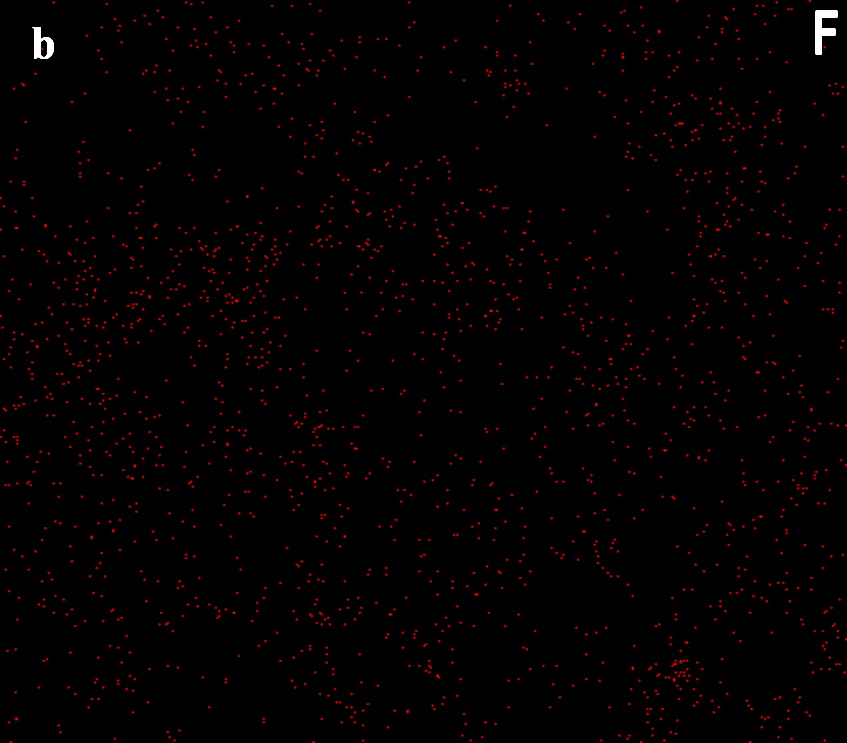

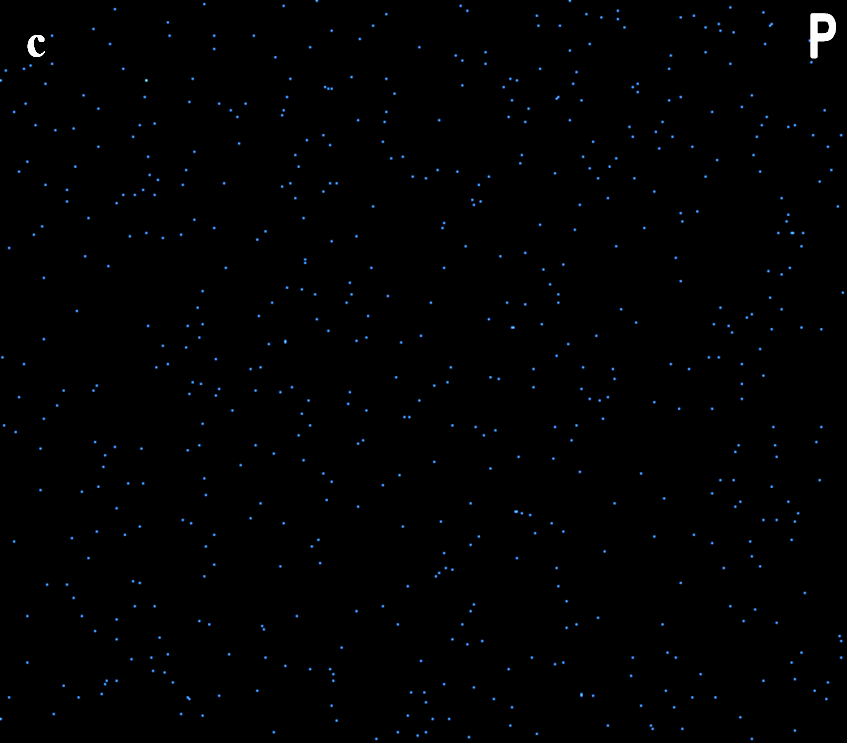

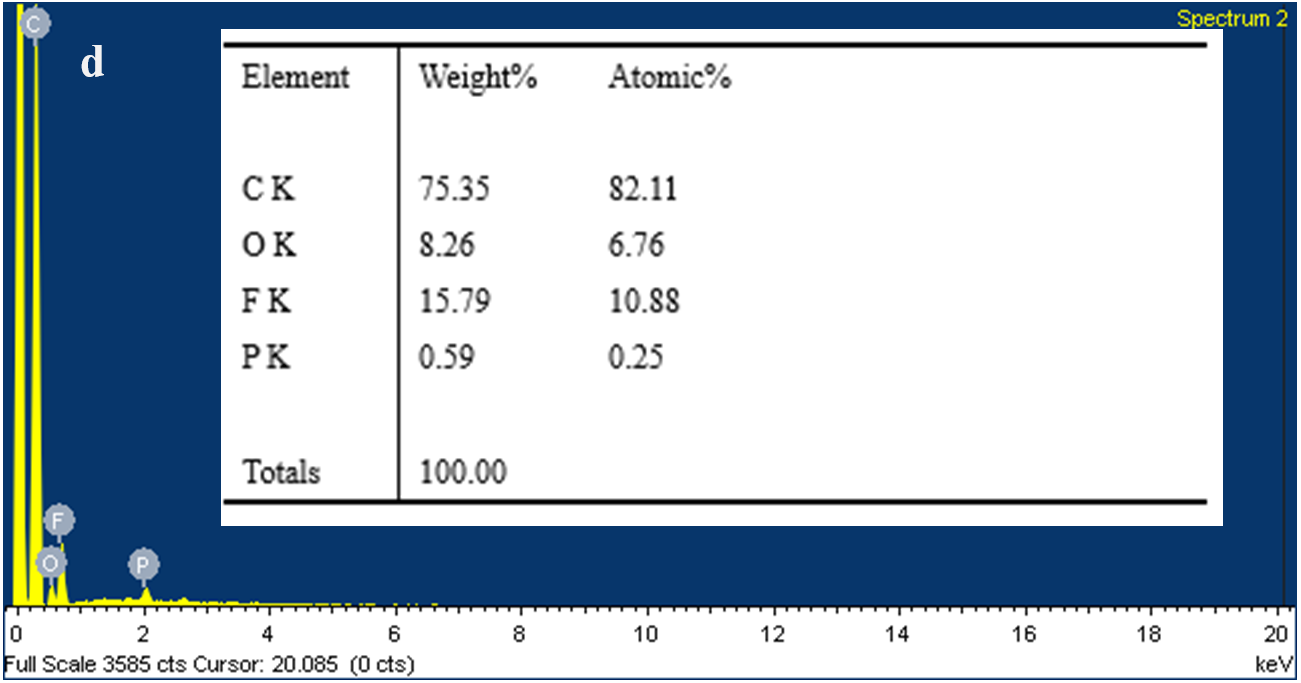


**Figure S8.** **Element mapping images and EDX spectrum for FLGC tape-cast electrode held at a discharge stage of 1.55 V in the Li-PF_6_ relay battery.** **a-c**) SEM and element mapping images, **d**) the corresponding EDX spectrum.


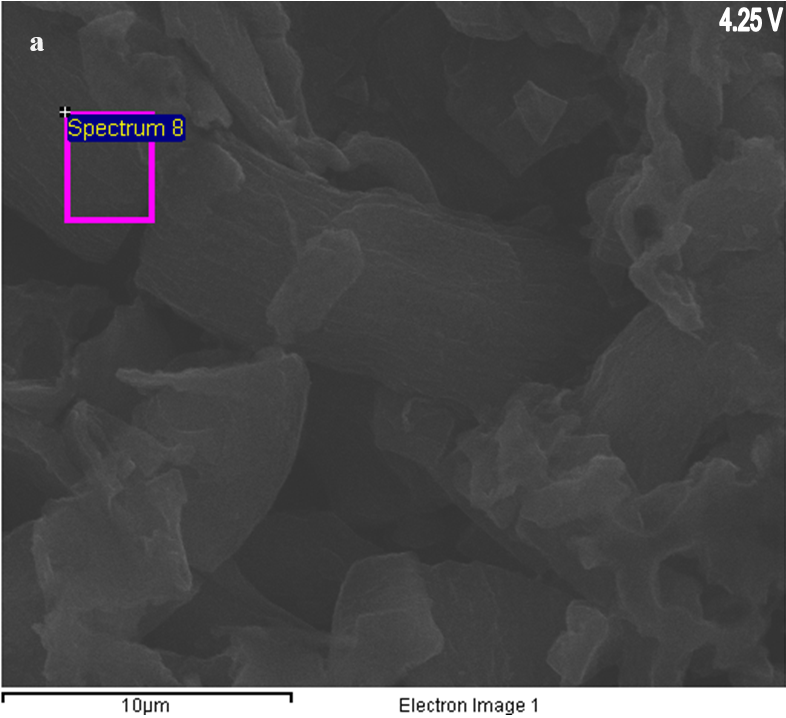

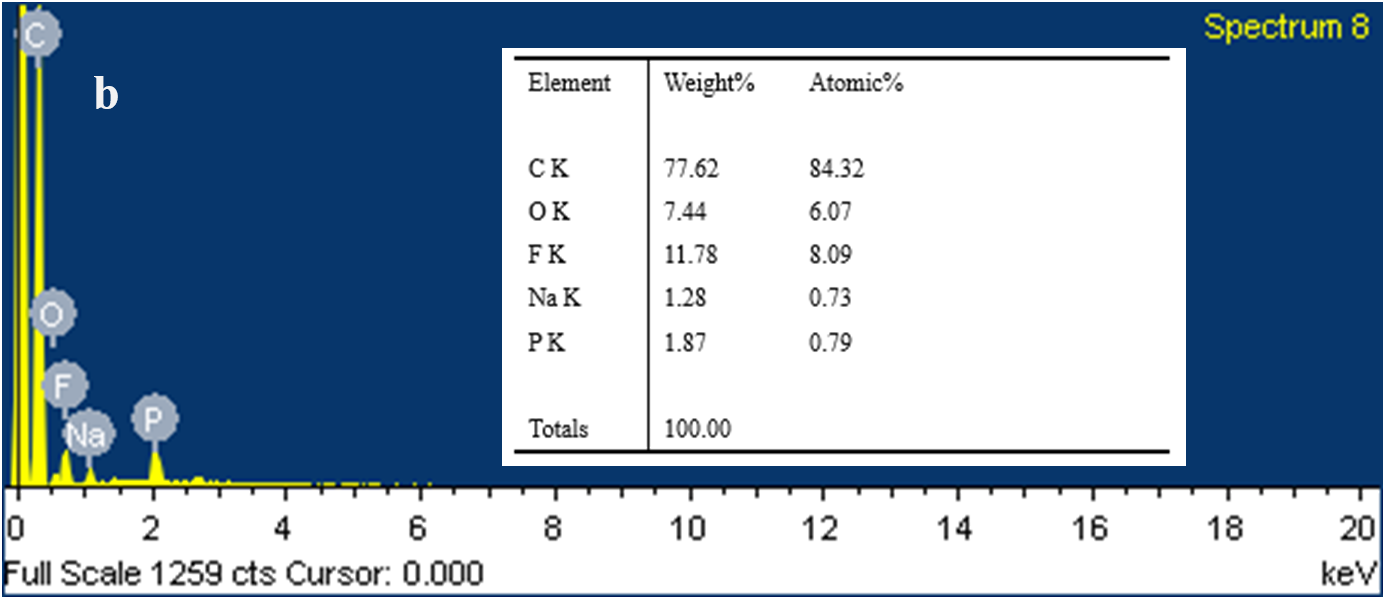


**Figure S9.** **FLGC tape-cast electrode held at a charge stage of 4.25 V in the Na-PF_6_ relay battery.**  **a**) SEM image, **b**) the corresponding EDX spectrum.


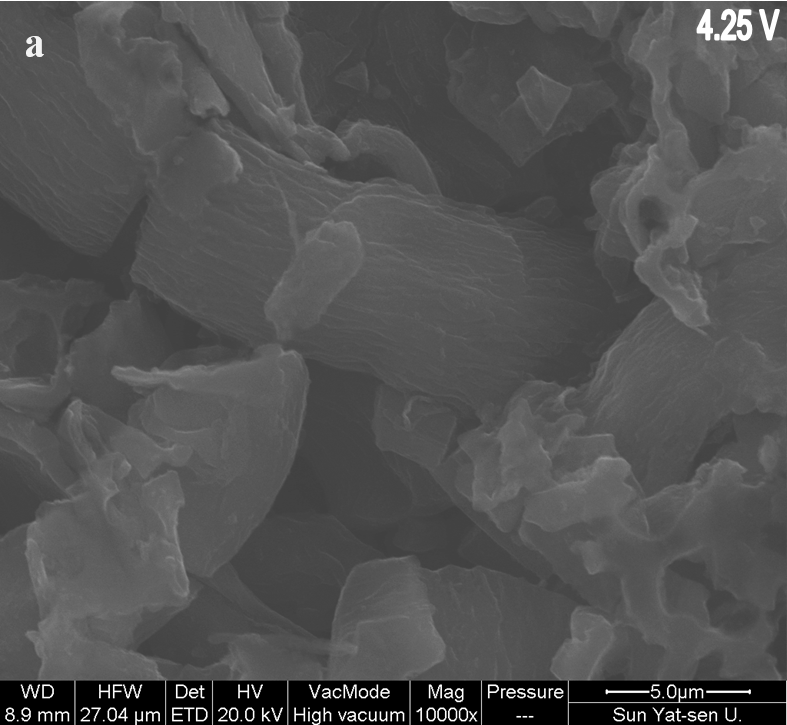

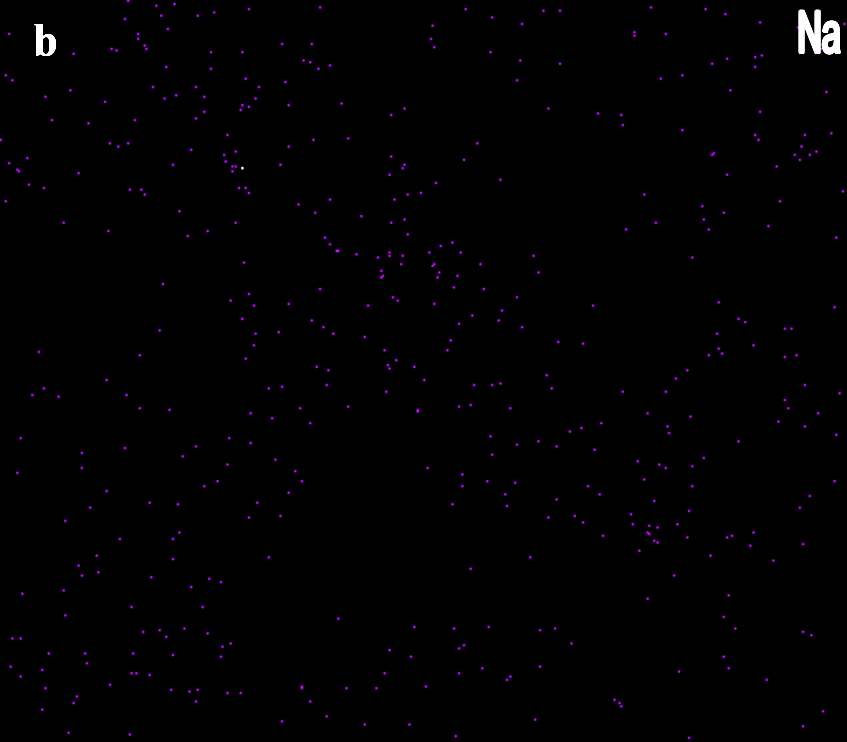

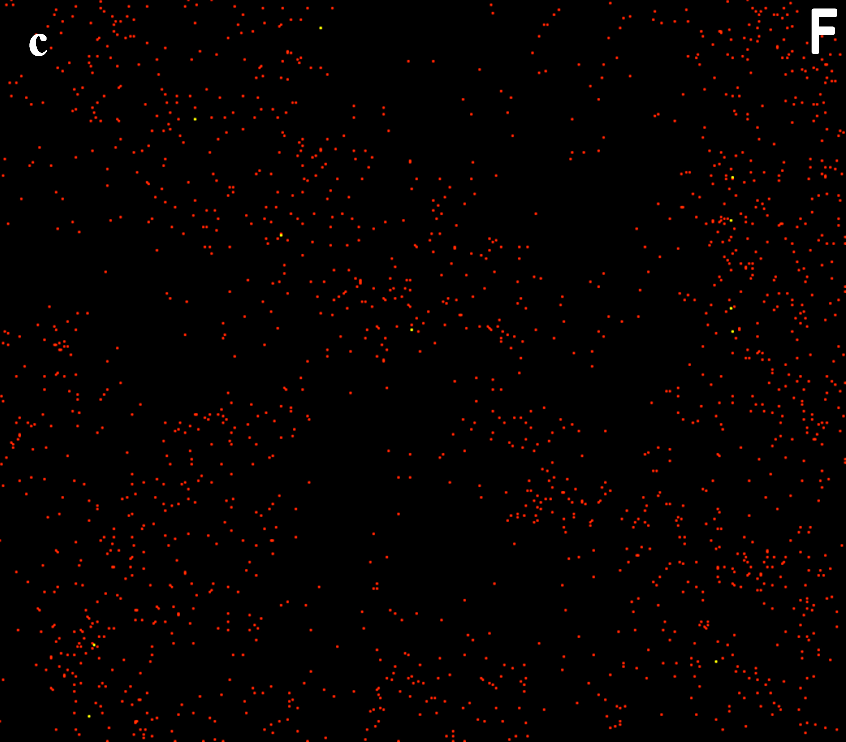

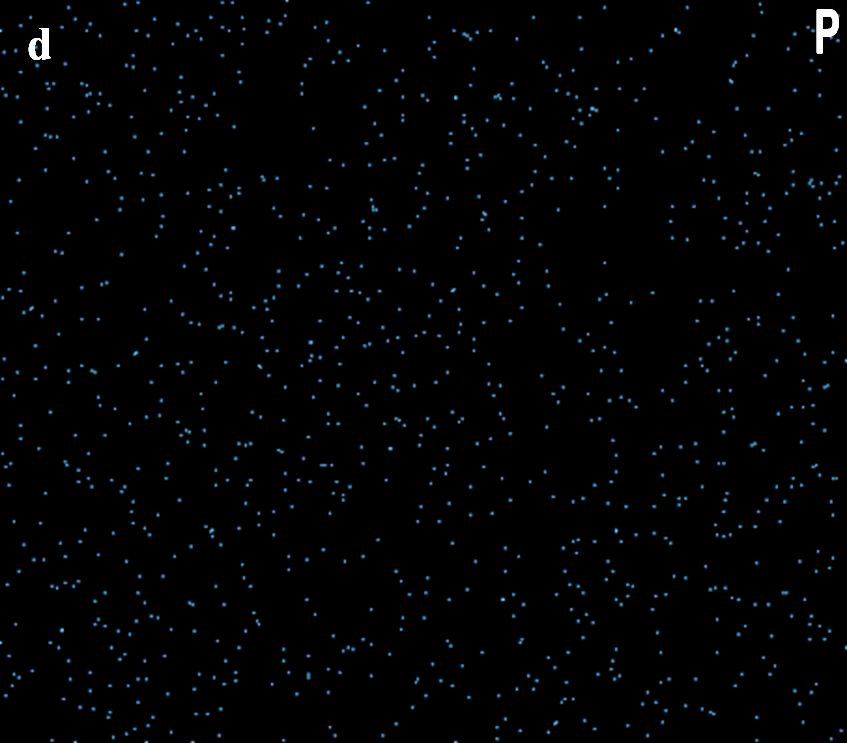


**Figure S10. The corresponding element mapping images for the sample presented in Fig.** S9**.**


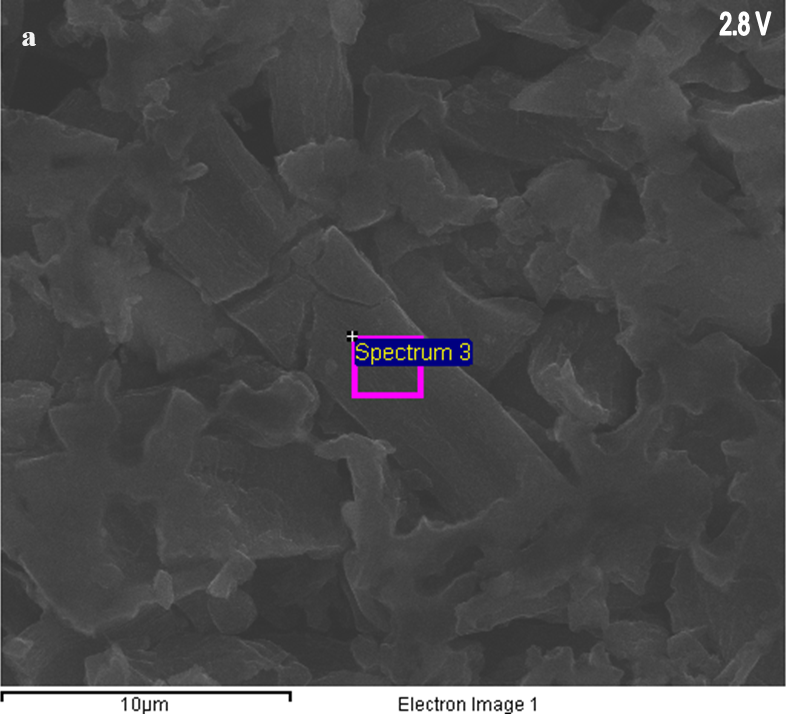

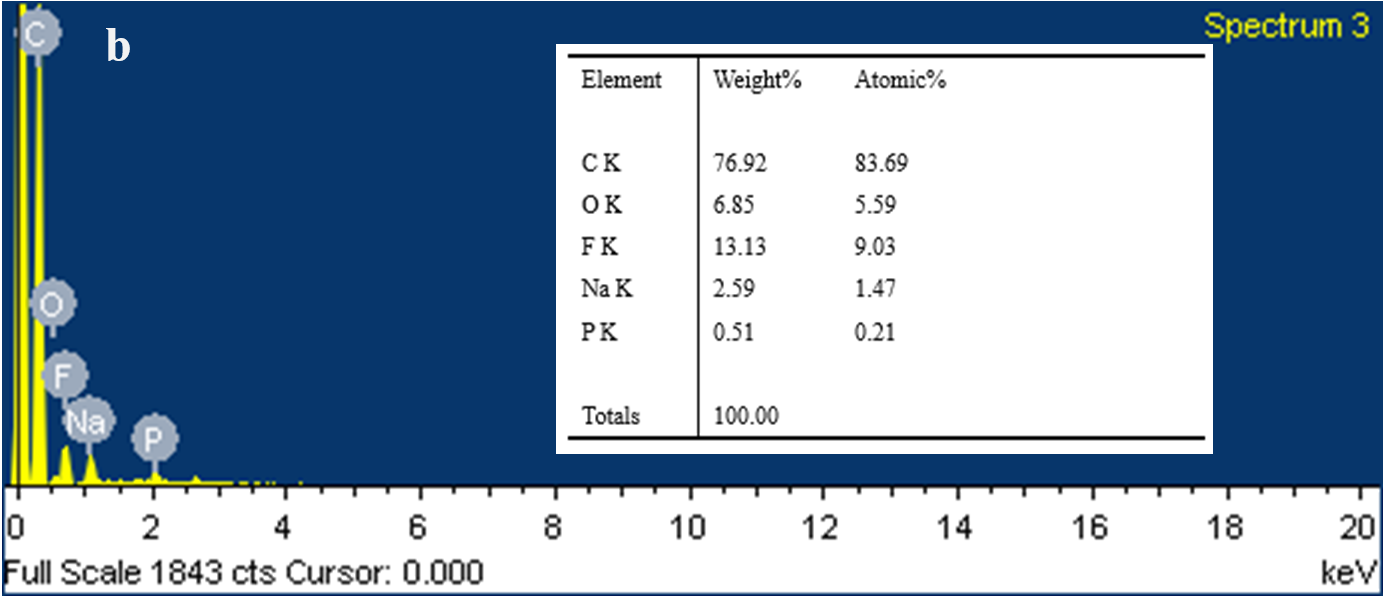


**Figure S11.** **FLGC tape-cast electrode held at a discharge stage of 2.8 V in the Na-PF_6_ relay battery.** **a**) SEM image, **b**) the corresponding EDX spectrum.


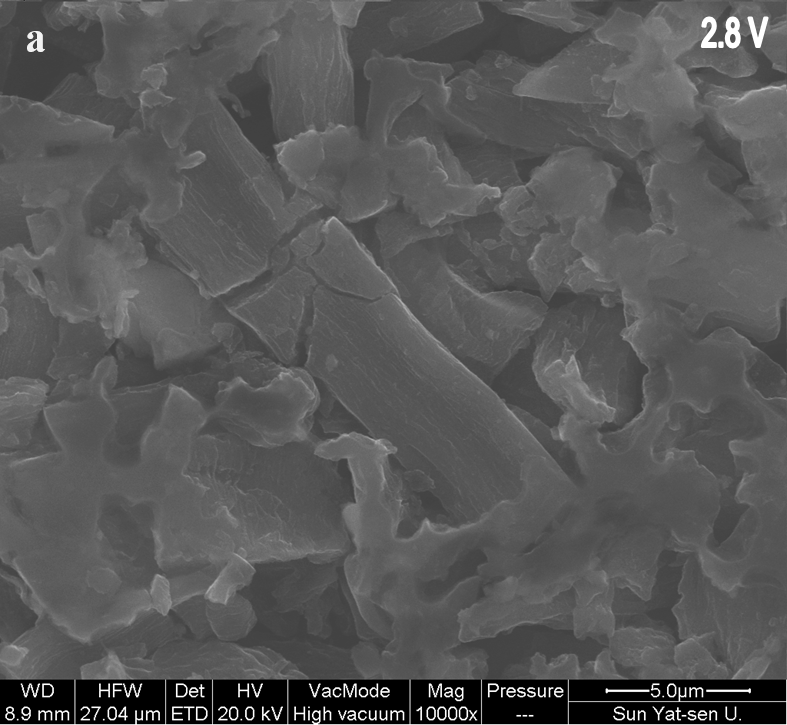

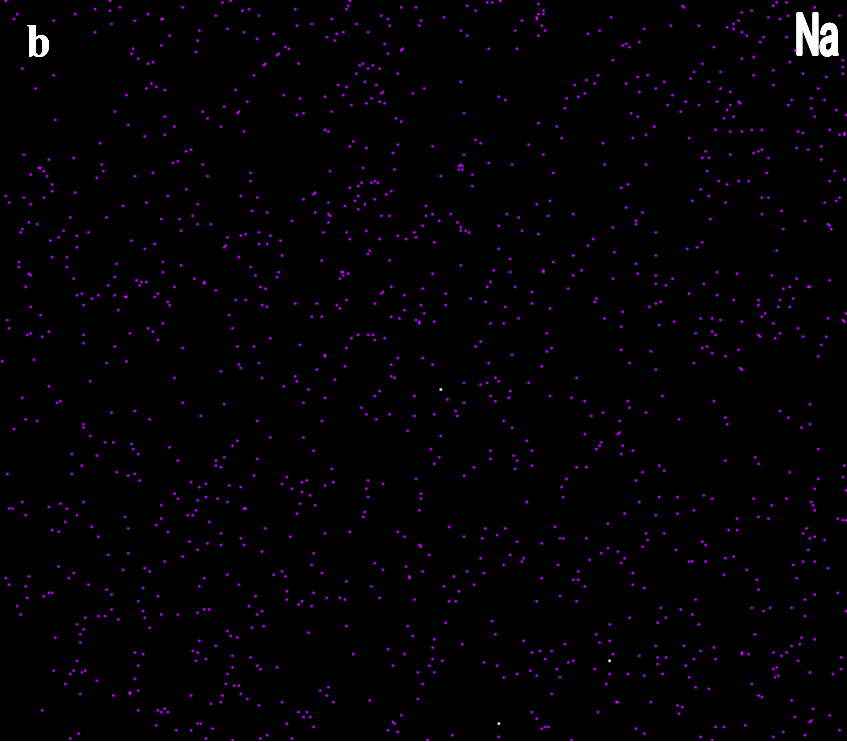

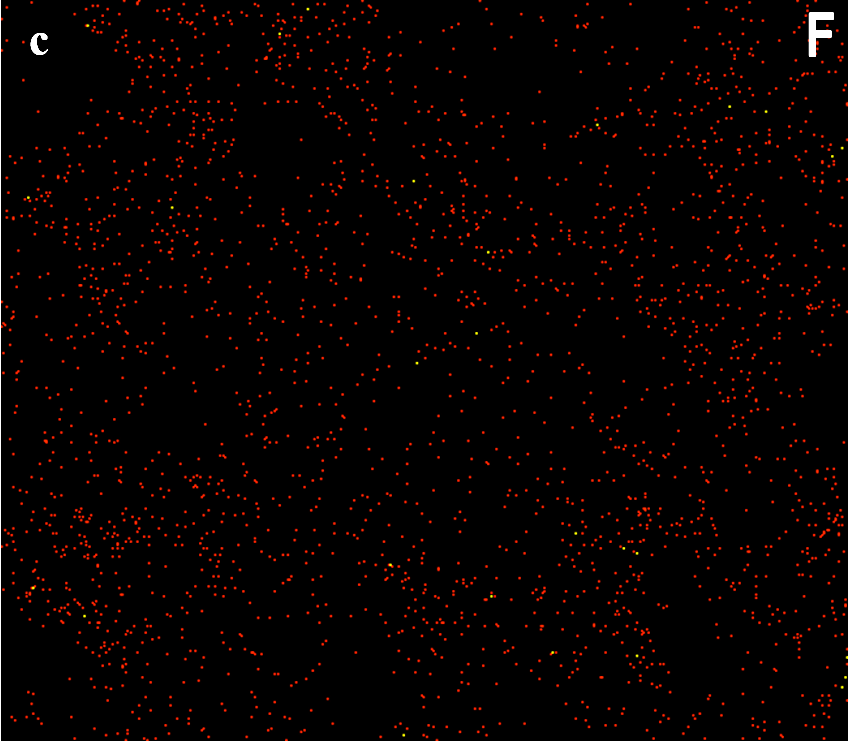

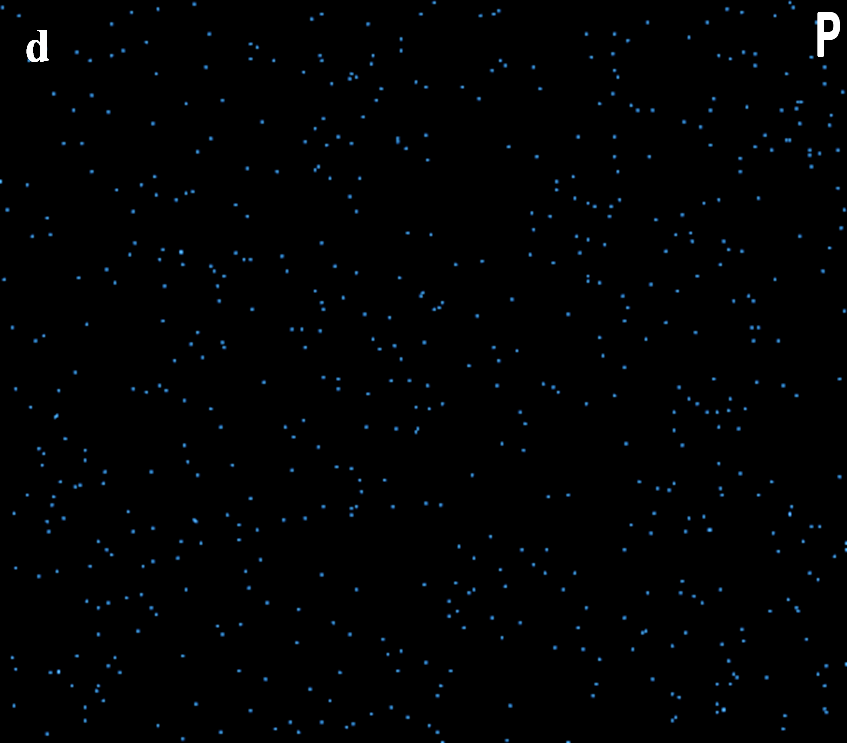


**Figure S12. The corresponding element mapping images for the sample presented in Fig.** S11**.**


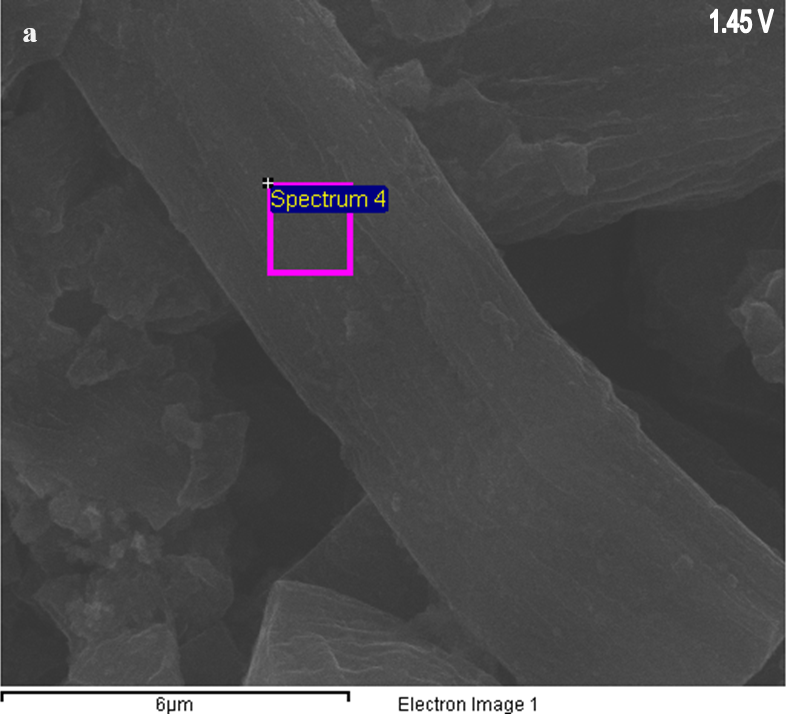

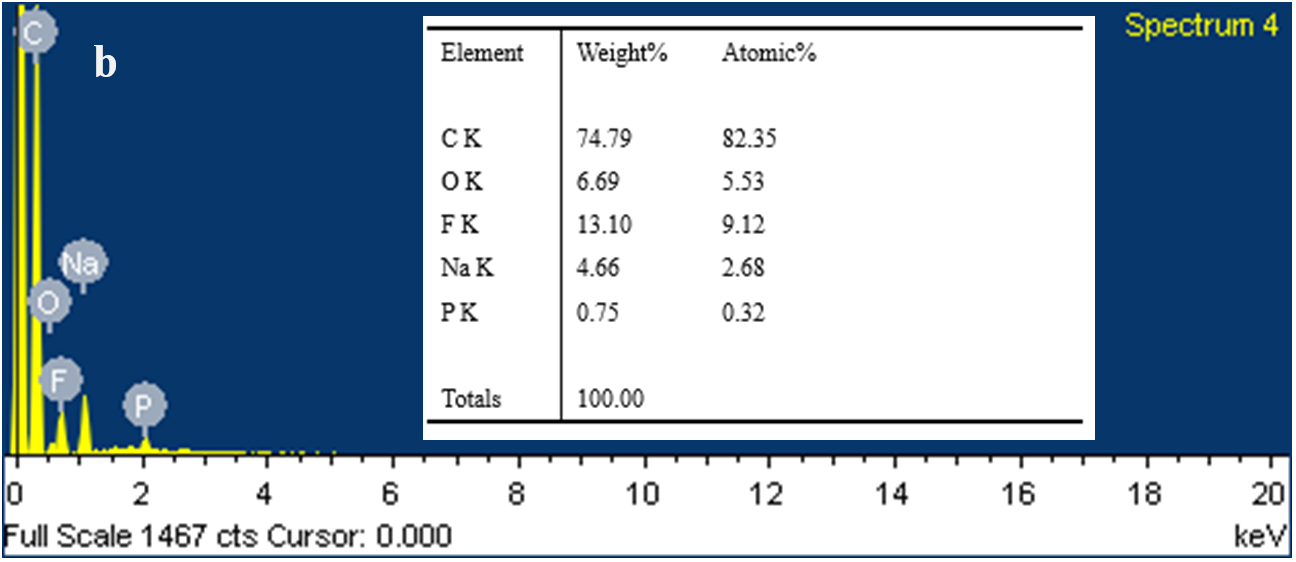


**Figure S13. FLGC tape-cast electrode held at a discharge stage of 1.45 V in the Na-PF_6_ relay battery.** **a**) SEM image, **b**) the corresponding EDX spectrum.


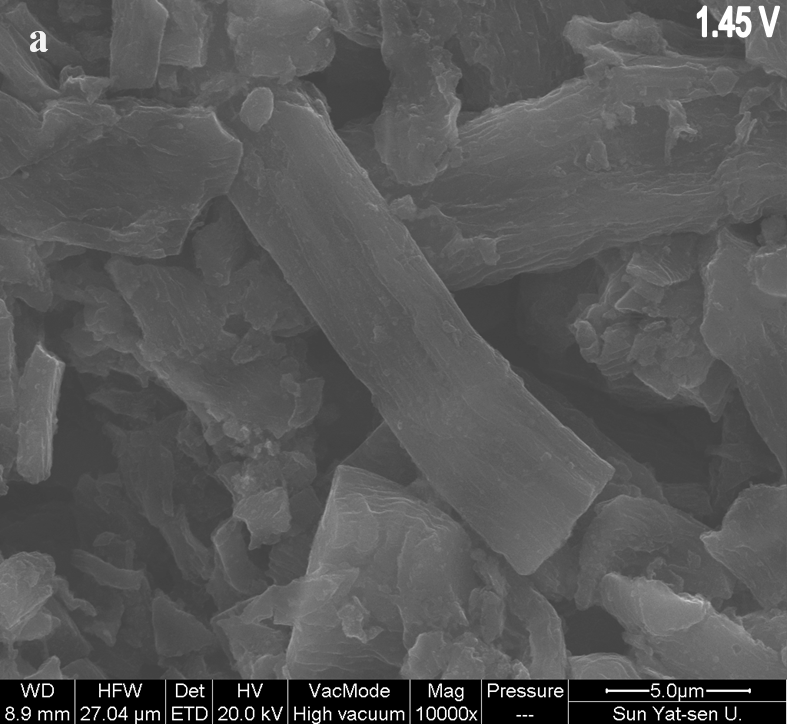

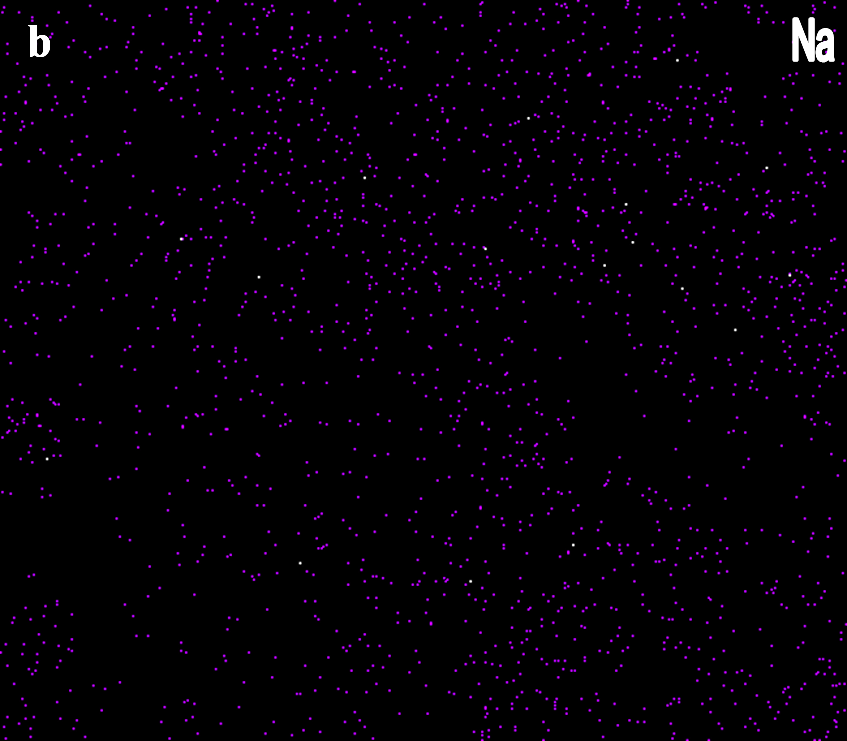

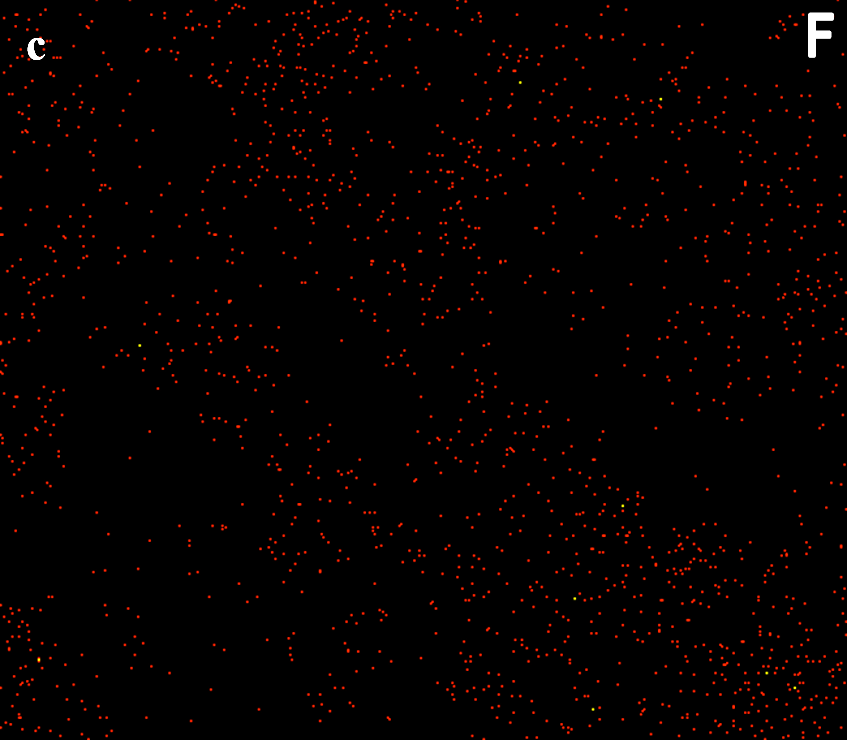

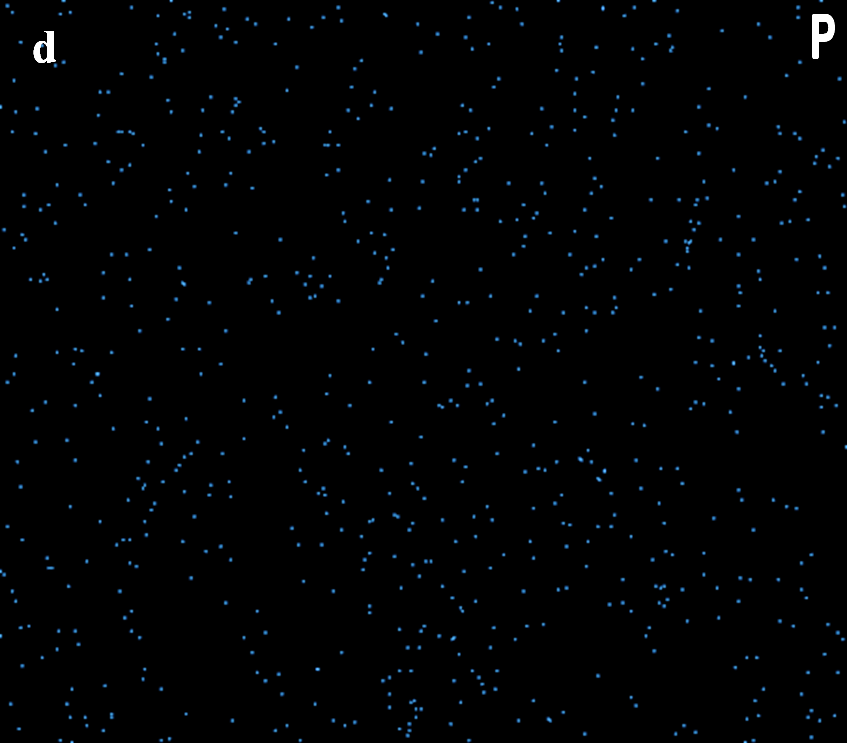


**Figure S14. The corresponding element mapping images for the sample presented in Fig.** S13**.**


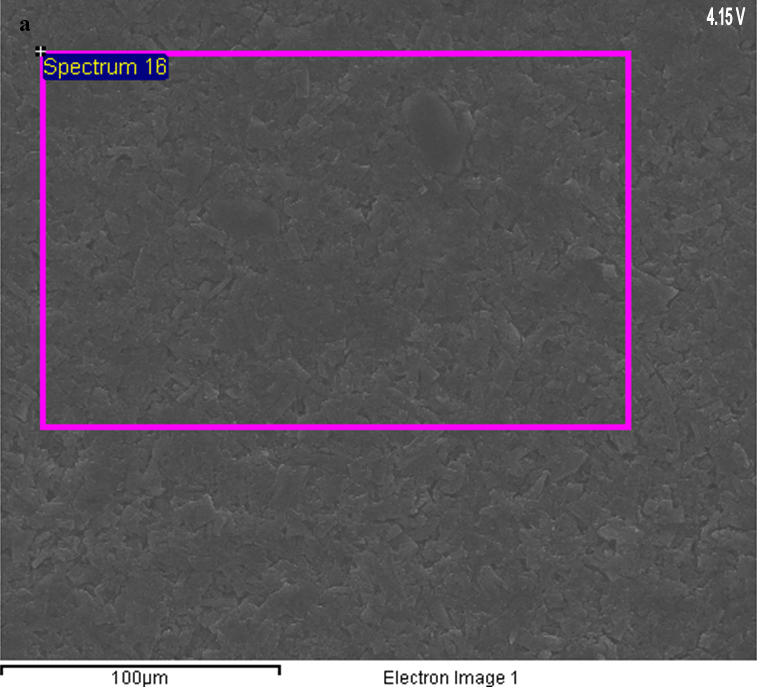

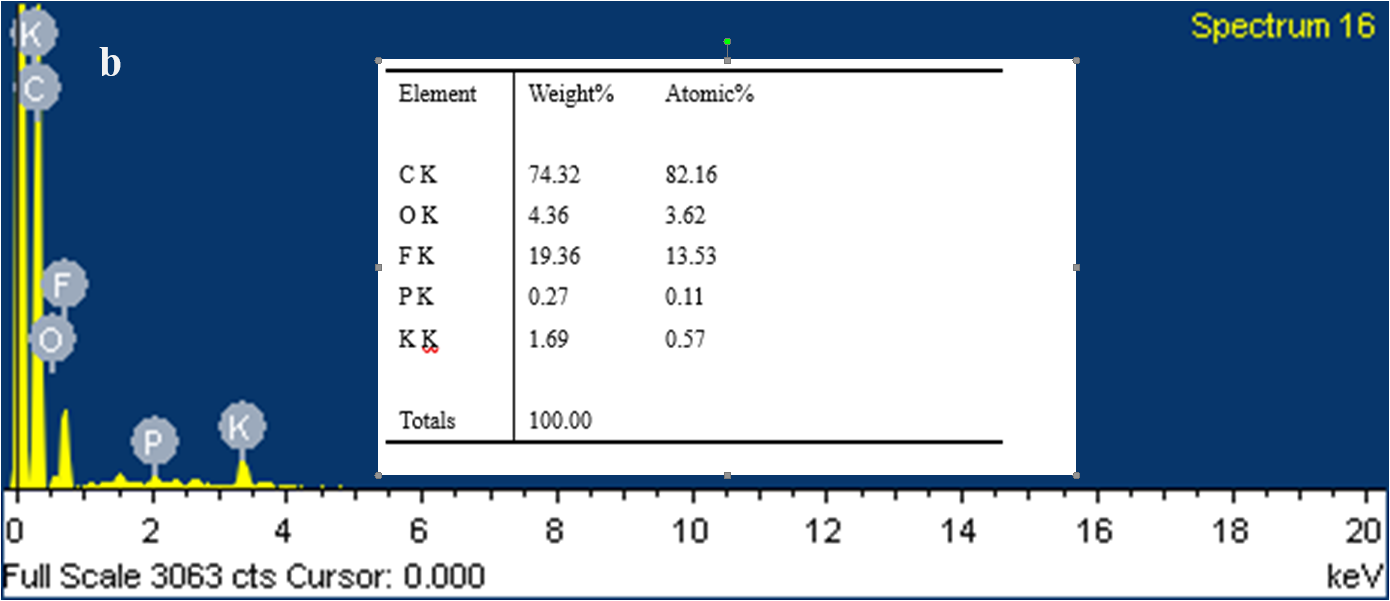


**Figure S15. FLGC tape-cast electrode held at a charge stage of 4.15 V in the K-PF_6_ relay battery. a**) SEM image, **b**) the corresponding EDX spectrum.


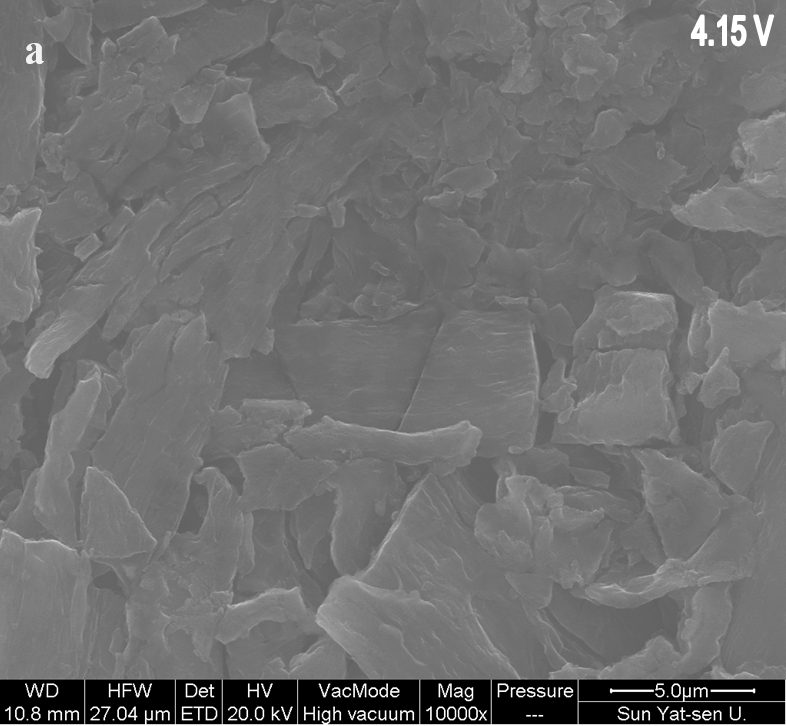

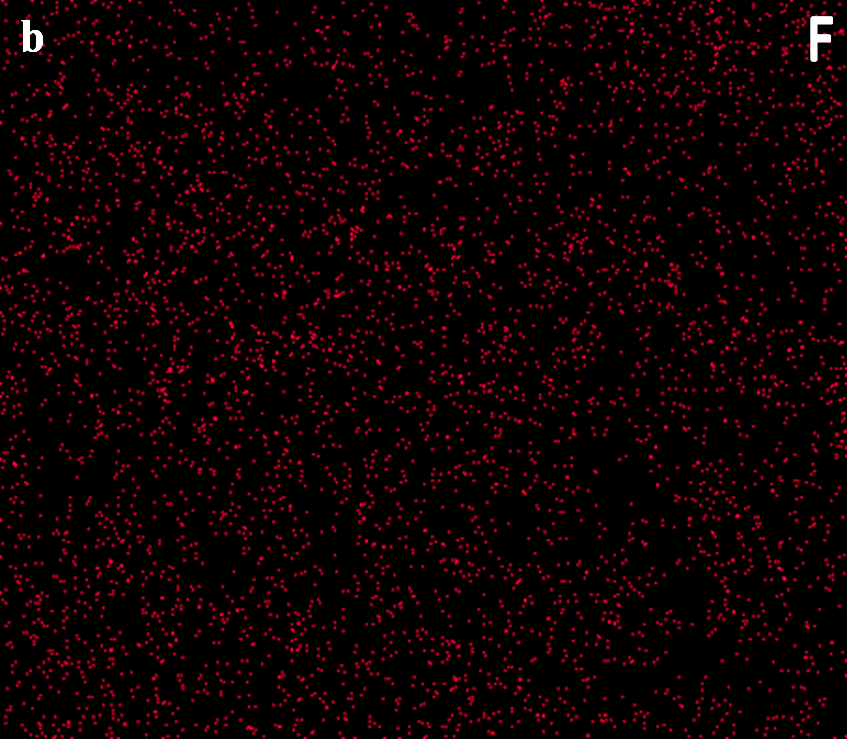

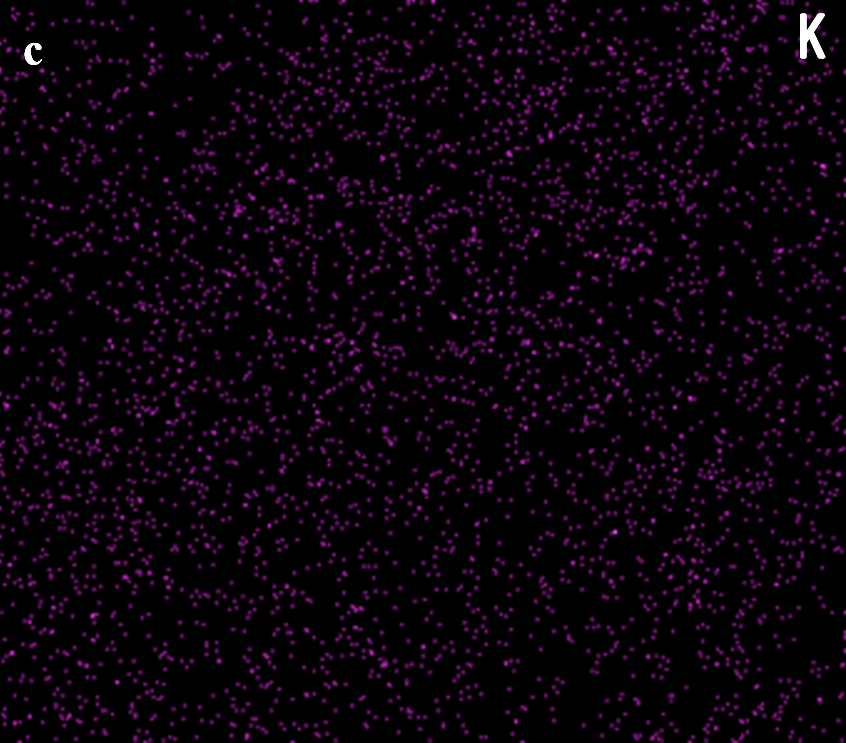

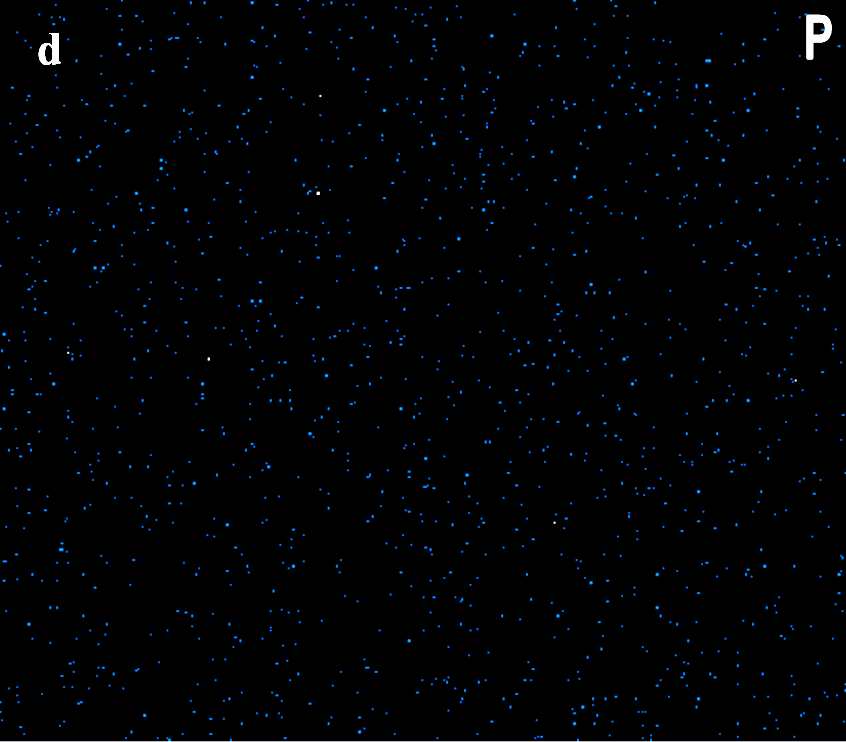


**Figure S16. The corresponding element mapping images for the sample presented in Fig.** S15**.**


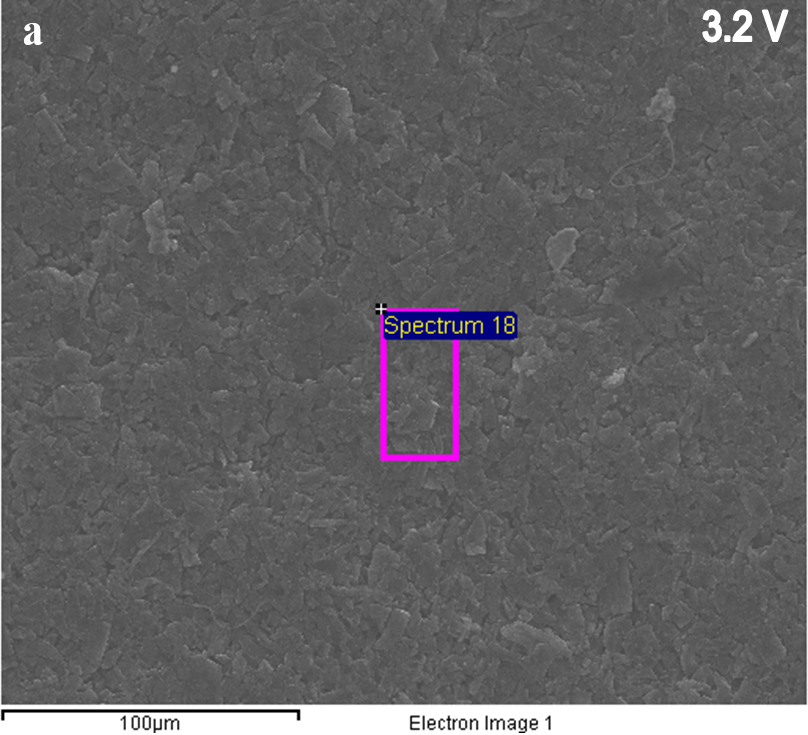

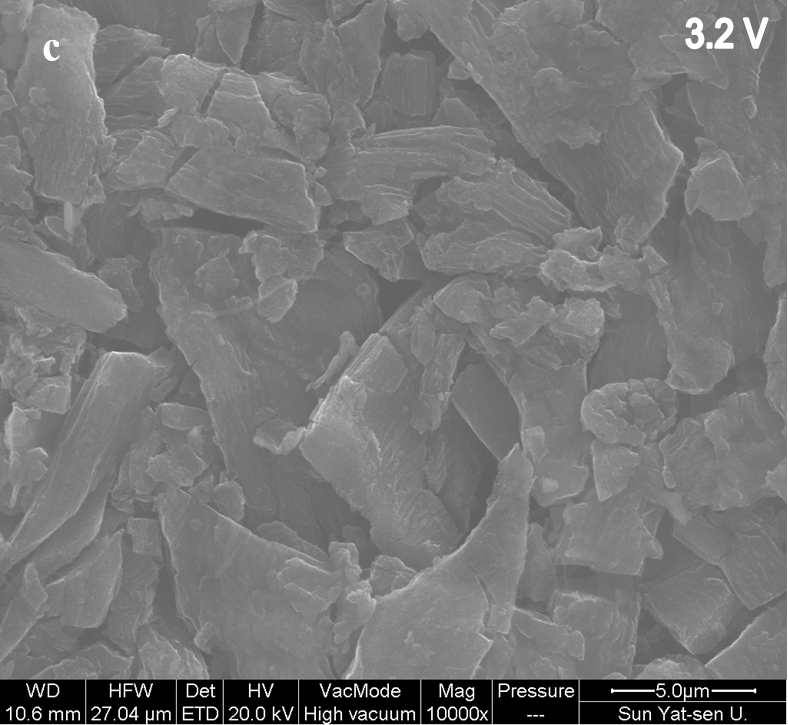
 **
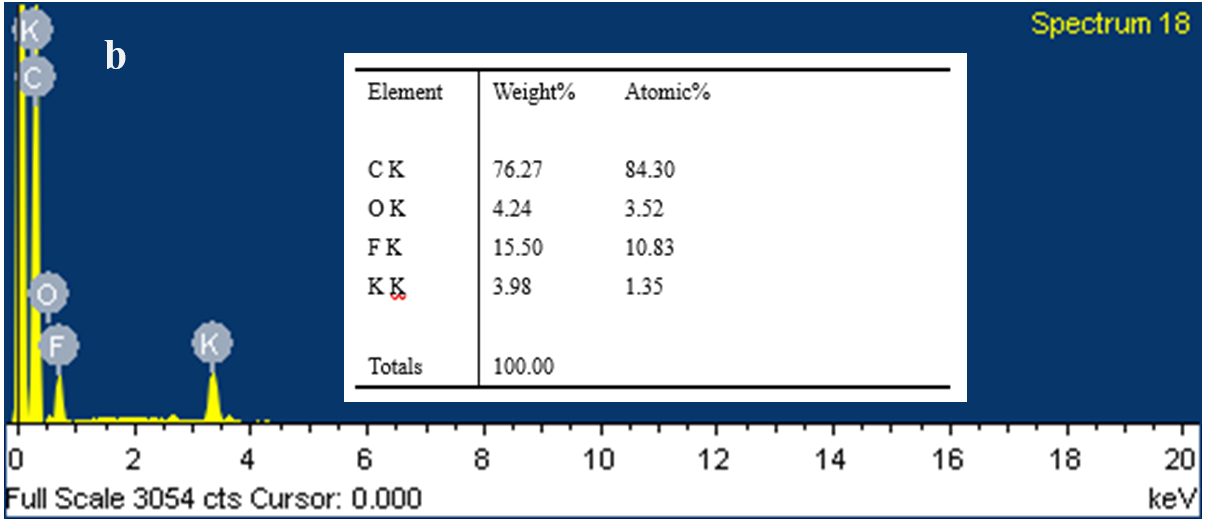
**
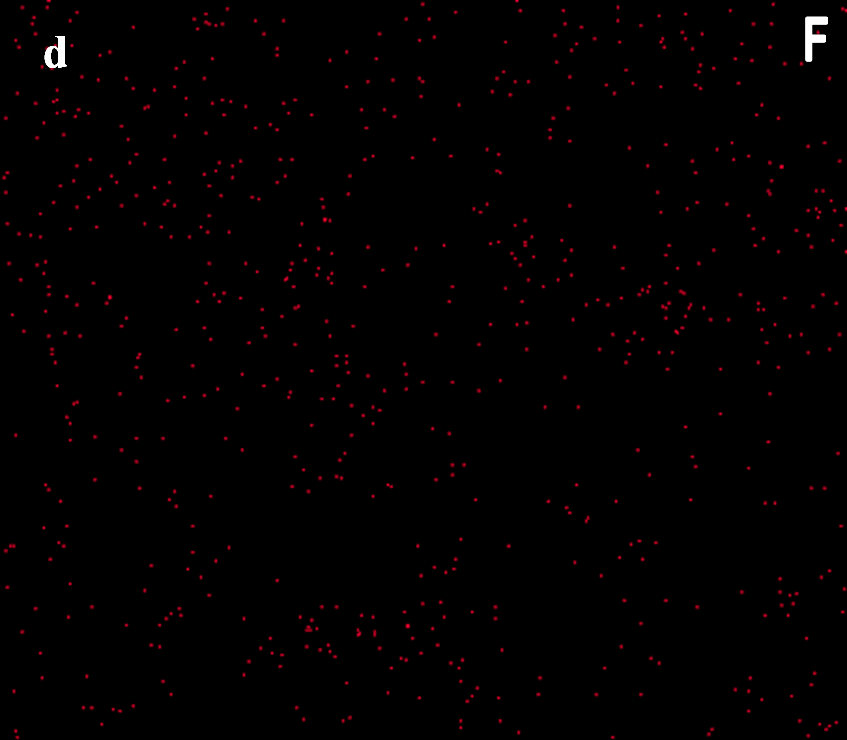

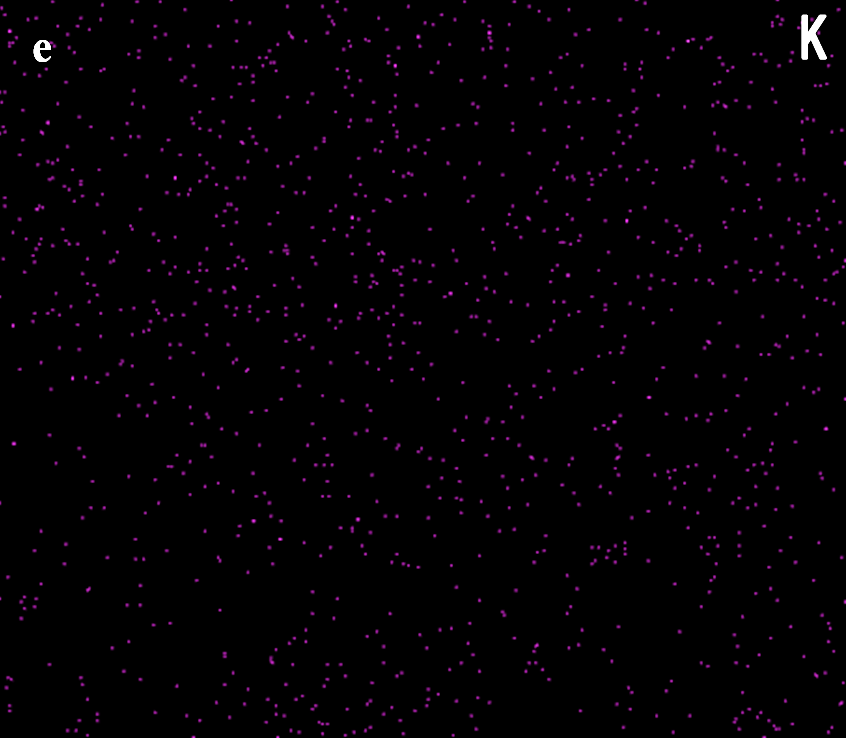


**Figure S17. FLGC tape-cast electrode held at a charge stage of 3.2 V in the K-PF_6_ relay battery.** **a**) SEM image, **b**) the corresponding EDX spectrum, **c-e**) the corresponding element mapping images.


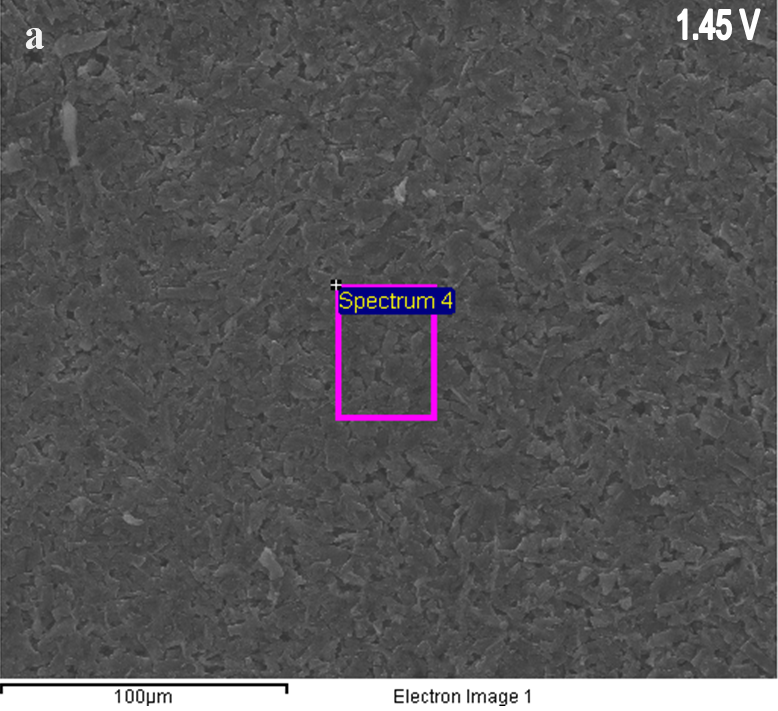

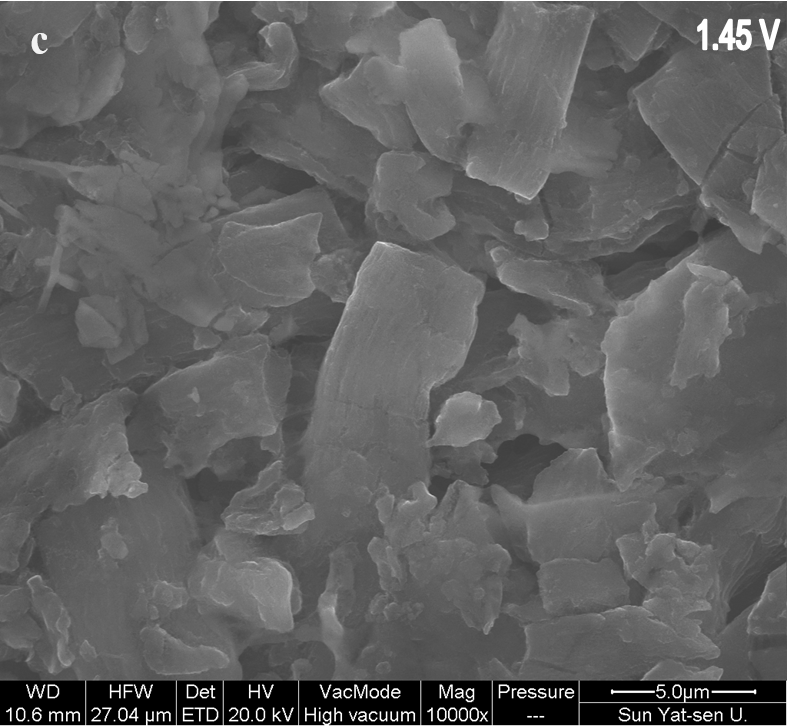

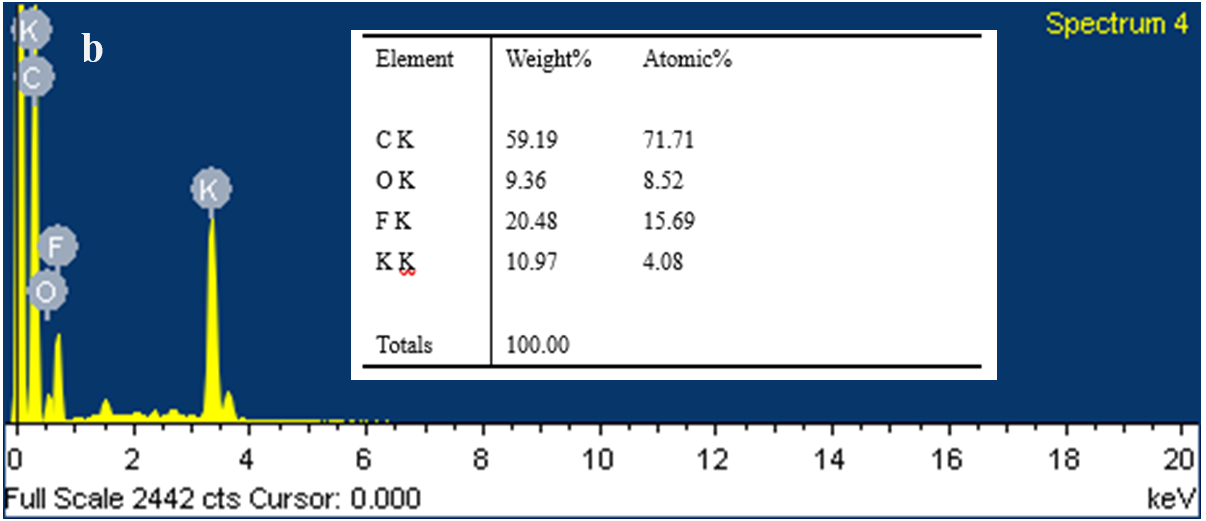

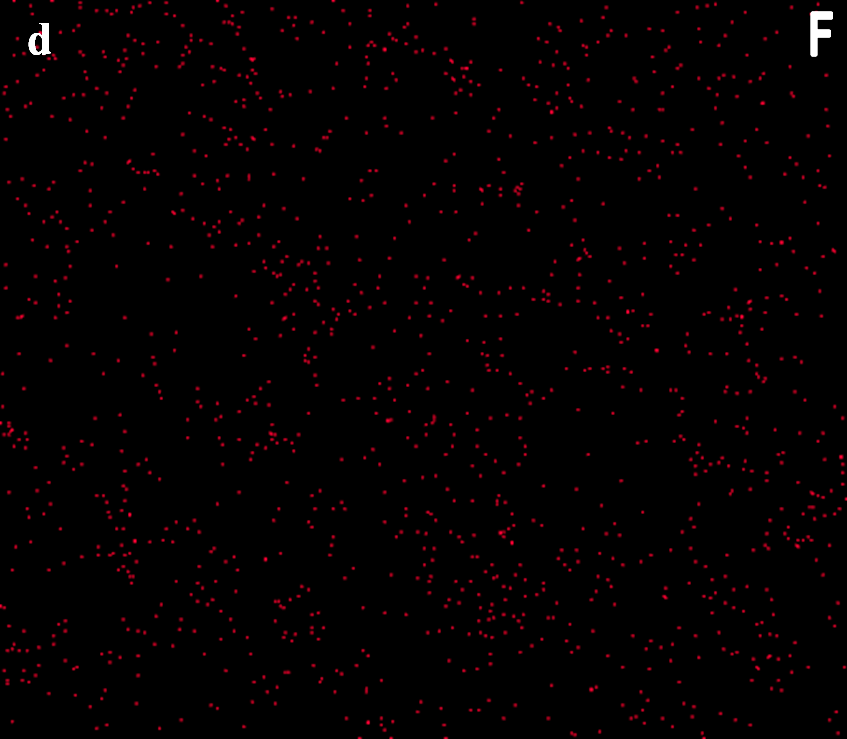

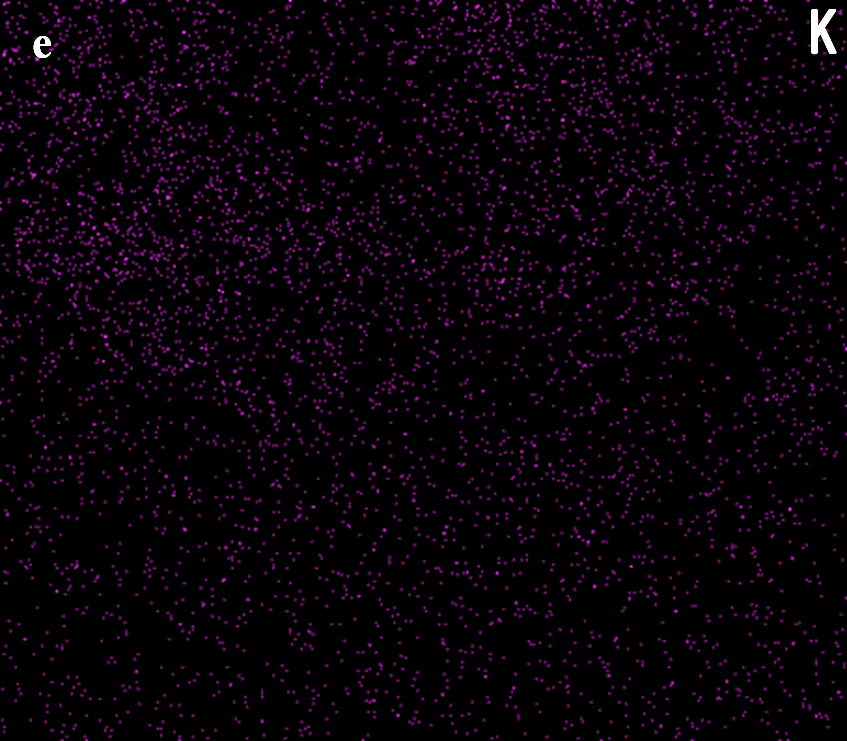


**Figure S18.** **FLGC tape-cast electrode held at a discharge stage of 1.45 V in the K-PF_6_ relay battery.** **a**) SEM image, **b**) the corresponding EDX spectrum, **c-e**) the corresponding element mapping images.


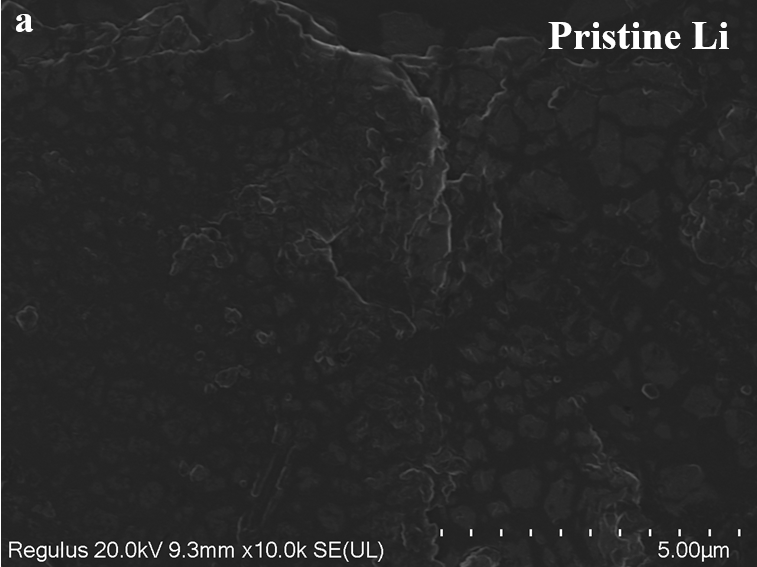

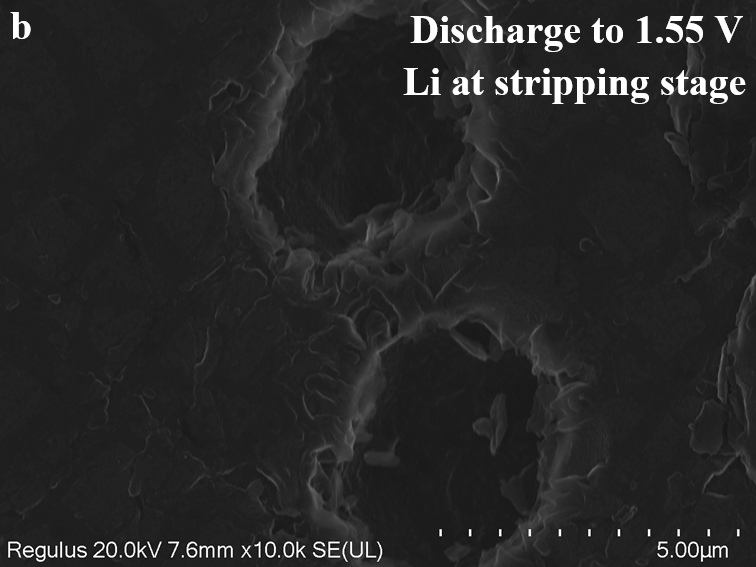


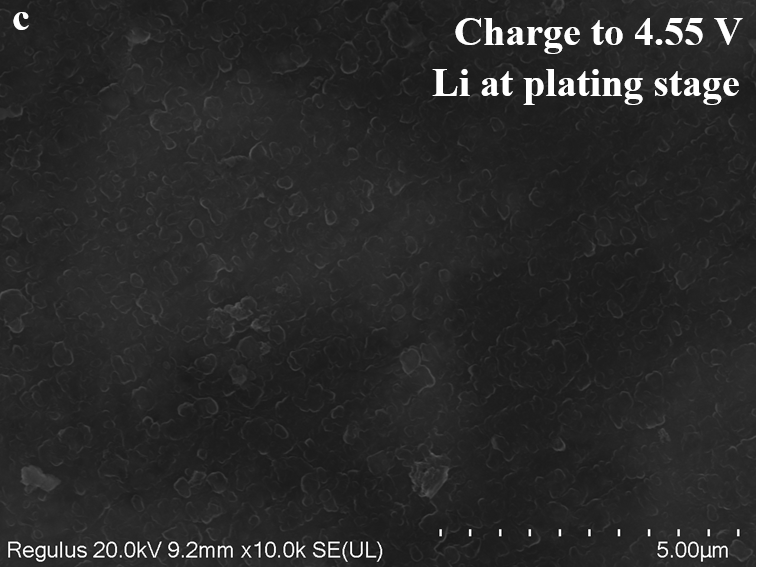


**Figure S19** **SEM images of Li anodes.** **a**) pristine, **b**) at stripping stage with the Li-PF_6_ ACRB discharged to 1.55 V, **c**) at plating stage with the Li-PF_6_ ACRB charged to 4.55 V.


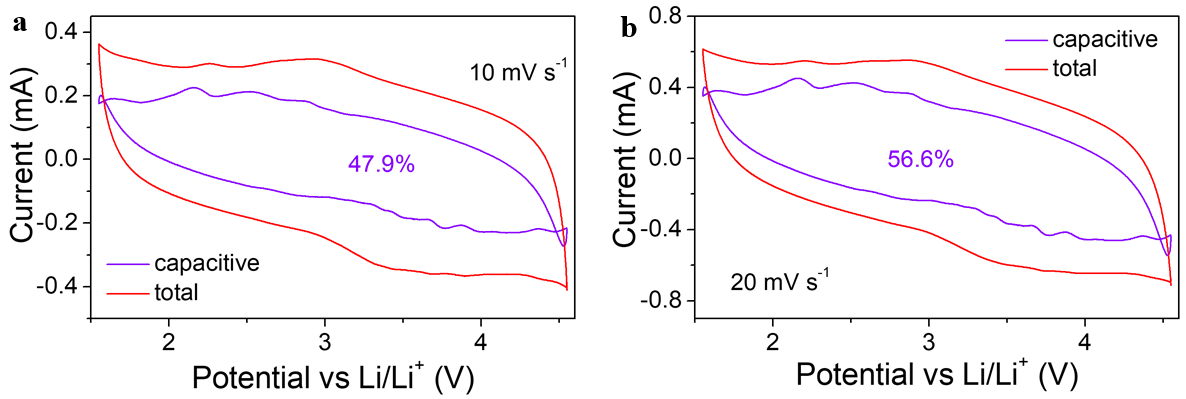

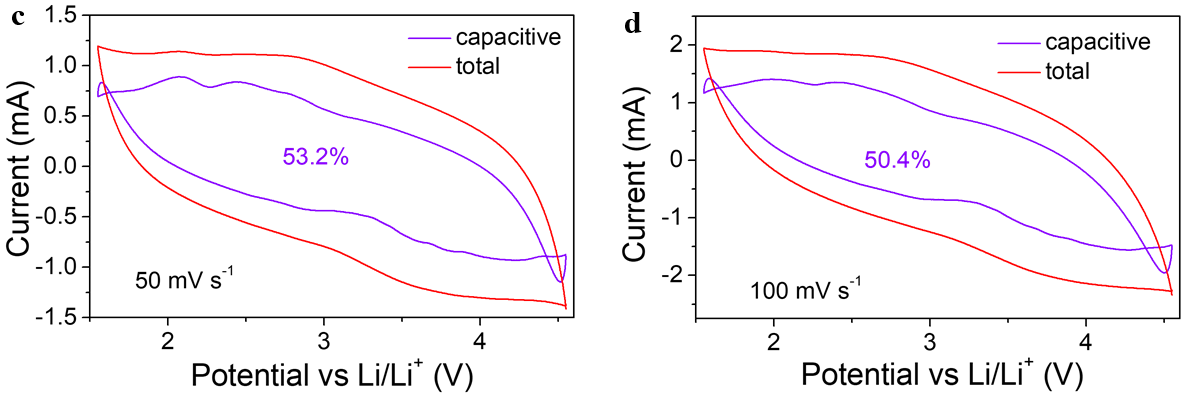


**Figure S20.** **Capacity contribution (capacitive or diffusion-controlled) at various scan rates for the Li-PF_6_ relay battery analyzed by sweep voltammetry according to previous reports**^1^**.** **a**) 10 mV s^-1^, **b**) 20 mV s^-1^, **c**) 50 mV s^-1^, **d**) 100 mV s^-1^.


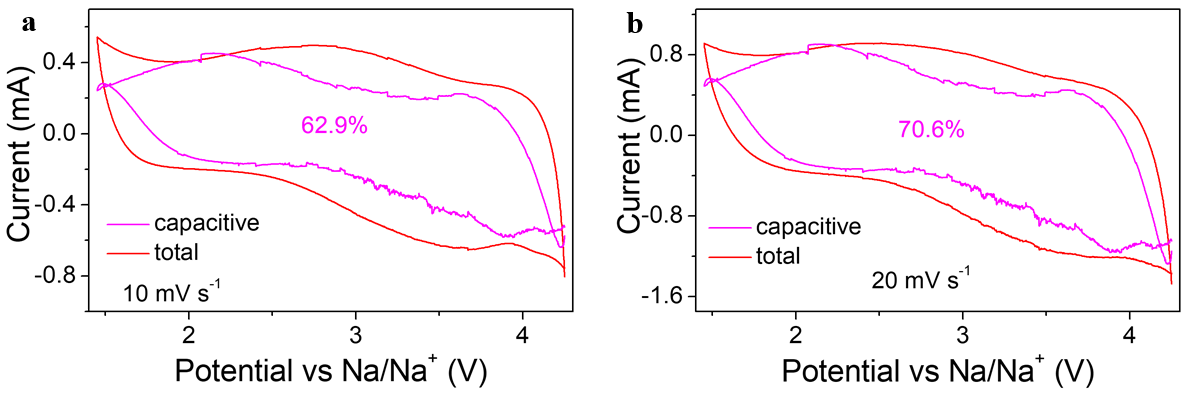

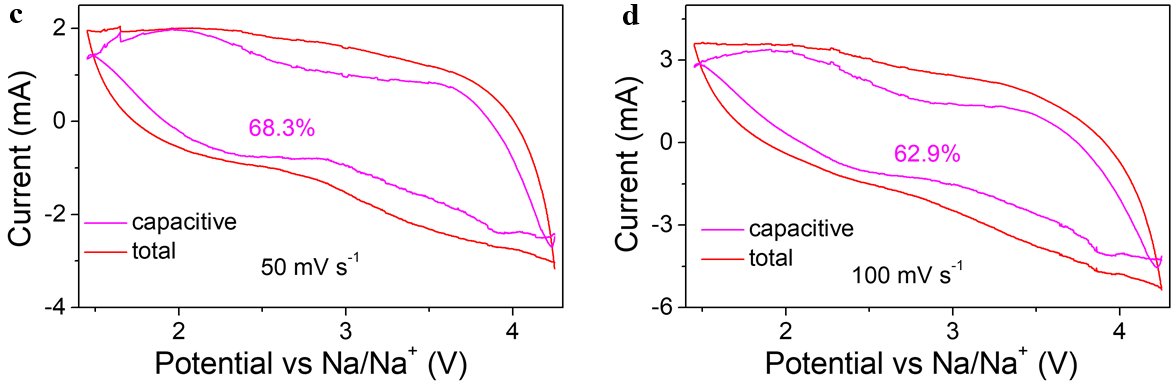


**Figure S21.** **Capacity contribution (capacitive or diffusion-controlled) at various scan rates for the Na-PF_6_ relay battery analyzed by sweep voltammetry according to previous reports**^1^**.** **a**) 10 mV s^-1^, **b**) 20 mV s^-1^, **c**) 50 mV s^-1^, **d**) 100 mV s^-1^.


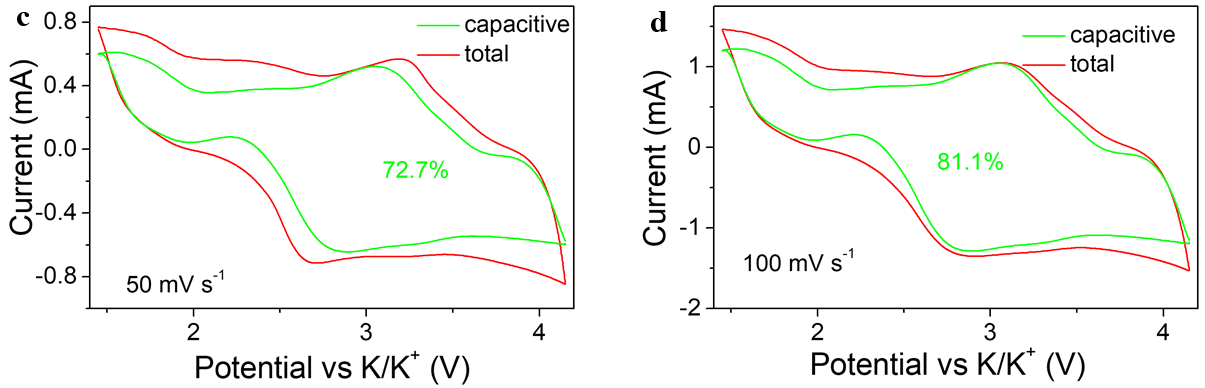

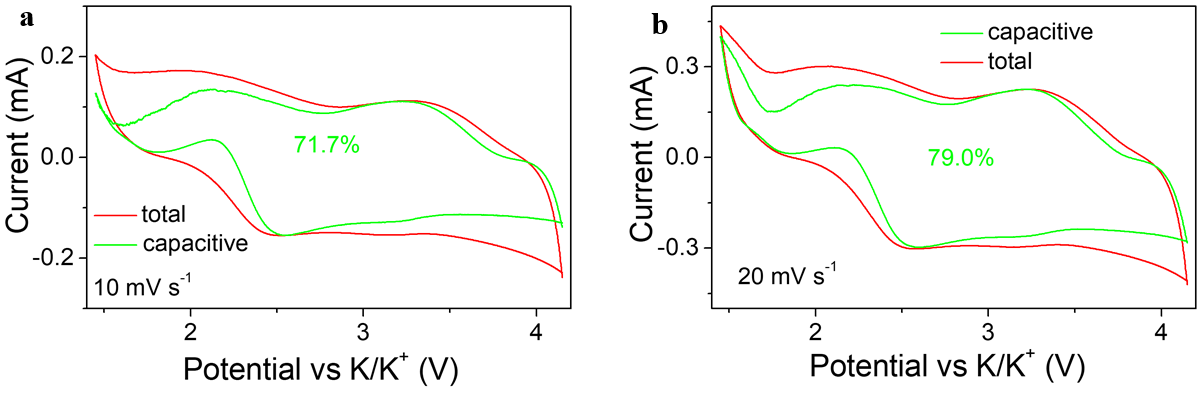


**Figure S22.** **Capacity contribution (capacitive or diffusion-controlled) at various scan rates for the K-PF_6_ relay battery analyzed by sweep voltammetry according to previous reports**^1^**.** **a**) 10 mV s^-1^, **b**) 20 mV s^-1^, **c**) 50 mV s^-1^, **d**) 100 mV s^-1^.

**References**

1. Brezesinski, T., Wang, J., Tolbert, S. H. & Dunn, B. Ordered mesoporous α-MoO_3_ with iso-oriented nanocrystalline walls for thin-film pseudocapacitors. *Nat. Mater.* **9**, 146-151 (2010).
